# PMO-based let-7c site blocking oligonucleotide (SBO) mediated utrophin upregulation in *mdx* mice, a therapeutic approach for Duchenne muscular dystrophy (DMD)

**DOI:** 10.1038/s41598-020-76338-1

**Published:** 2020-12-09

**Authors:** Kasturi Sengupta, Emanuele Loro, Tejvir S. Khurana

**Affiliations:** Department of Physiology and Pennsylvania Muscle Institute, Perelman School of Medicine, University of Pennsylvania, 755 Clinical Research Building, Philadelphia, PA 19104 USA

**Keywords:** Target validation, Neuromuscular disease

## Abstract

Upregulation of utrophin, a dystrophin related protein, is considered a promising therapeutic approach for Duchenne muscular dystrophy (DMD). Utrophin expression is repressed at the post-transcriptional level by a set of miRNAs, among which let-7c is evolutionarily highly conserved. We designed PMO-based SBOs complementary to the let-7c binding site in *UTRN* 3′UTR, with the goal of inhibiting let-7c interaction with *UTRN* mRNA and thus upregulating utrophin. We used the C2C12*UTRN*5′luc3′ reporter cell line in which the 5′- and 3′-UTRs of human *UTRN* sequences flank luciferase, for reporter assays and the C2C12 cell line for utrophin western blots, to independently evaluate the site blocking efficiency of a series of let-7c PMOs in vitro. Treatment of one-month old *mdx* mice with the most effective let-7c PMO (i.e. S56) resulted in ca. two-fold higher utrophin protein expression in skeletal muscles and the improvement in dystrophic pathophysiology in *mdx* mice, in vivo. In summary, we show that PMO-based let-7c SBO has potential applicability for upregulating utrophin expression as a therapeutic approach for DMD.

## Introduction

DMD is a lethal muscle wasting disease affecting approximately 1 in ~ 5000 live-born males worldwide^1,2^. DMD is caused by mutations in the *DMD* gene resulting in complete loss or extremely low expression of the dystrophin protein^3,4^. Dystrophin (427 kDa) links the cytoskeleton with the extracellular matrix and is a key component of the dystrophin glycoprotein complex (DGC)^5–7^. During muscle contraction and relaxation cycles, dystrophin provides structural integrity to the myofiber^8^. Loss of dystrophin and the concomitant destabilization of DGC, is thought to result in sarcolemmal fragility^9^, altered membrane protein related signaling^10^ and defective muscle regeneration^11^. Increased muscle damage leads to cycles of abortive muscle degeneration and regeneration accompanied by chronic inflammation. Eventually, contractile units are replaced with fibro-fatty tissues leading to a severe degree of muscle weakness and wasting. The disease progression typically leads to loss of ambulation in the early teenage years and respiratory and cardiac failure by the fourth decade of life^12^.

A promising therapeutic approach for DMD is upregulating utrophin which is an autosomal paralog of dystrophin. Utrophin could functionally compensate for the lack of dystrophin as it has a high degree of structural and functional similarity^13–15^. The major utrophin isoform in myofibers, utrophin-A, is enriched at the neuromuscular and myotendinous junctions of adult muscles, and at the sarcolemma of regenerating myofibers^16–19^. Utrophin upregulation has been obtained with small molecules such as heregulin^20^, nabumetone^21^, SMT C1100^22^; artificial transcription factors^23,24^ and by regulating miRNAs that repress *UTRN* expression^25–27^. miRNAs are short (~ 22nt) noncoding RNAs which, in association with RNA induced silencing complex (RISC), target complementary binding sites mostly within 3′UTRs of mRNAs. miRNAs are involved in the post-transcriptional regulation of gene expression, typically by interfering with the stability and/or translation of the target mRNAs^28,29^. Previously, we have used 2′-*O*-methyl-phosphorothioate (2OMePS) SBOs to block the let-7c binding site in the *UTRN* 3′UTR and obtained upregulation of utrophin protein as well as functional improvement in *mdx* mice^30^. However, the chemical moiety has limitations as the charged nature of 2OMePS leads to suboptimal pharmacokinetic (PK) properties and therapeutic efficacy in terms of drug development^31^. Clinical trials with dystrophin exon 51 skipping 2OMePS antisense oligonucleotides (AONs) have reported improvements in DMD however, they were also accompanied by some side effects e.g. proteinuria and moderate thrombocytopenia, that hampered progress^32–34^.

Among the alternative chemistries, phosphorodiamidate morpholino oligonucleotides (PMOs), in which the phosphorodiamidate backbone is linked with a morpholino moiety, have lower affinity for plasma proteins, higher binding affinity for their target sequences and can achieve higher tissue concentrations^35,36^. Indeed, FDA has recently approved PMO-based AONs for exon 51 and exon 53 skipping in a small subset of DMD patients^37,38^.

**Figure 1:**
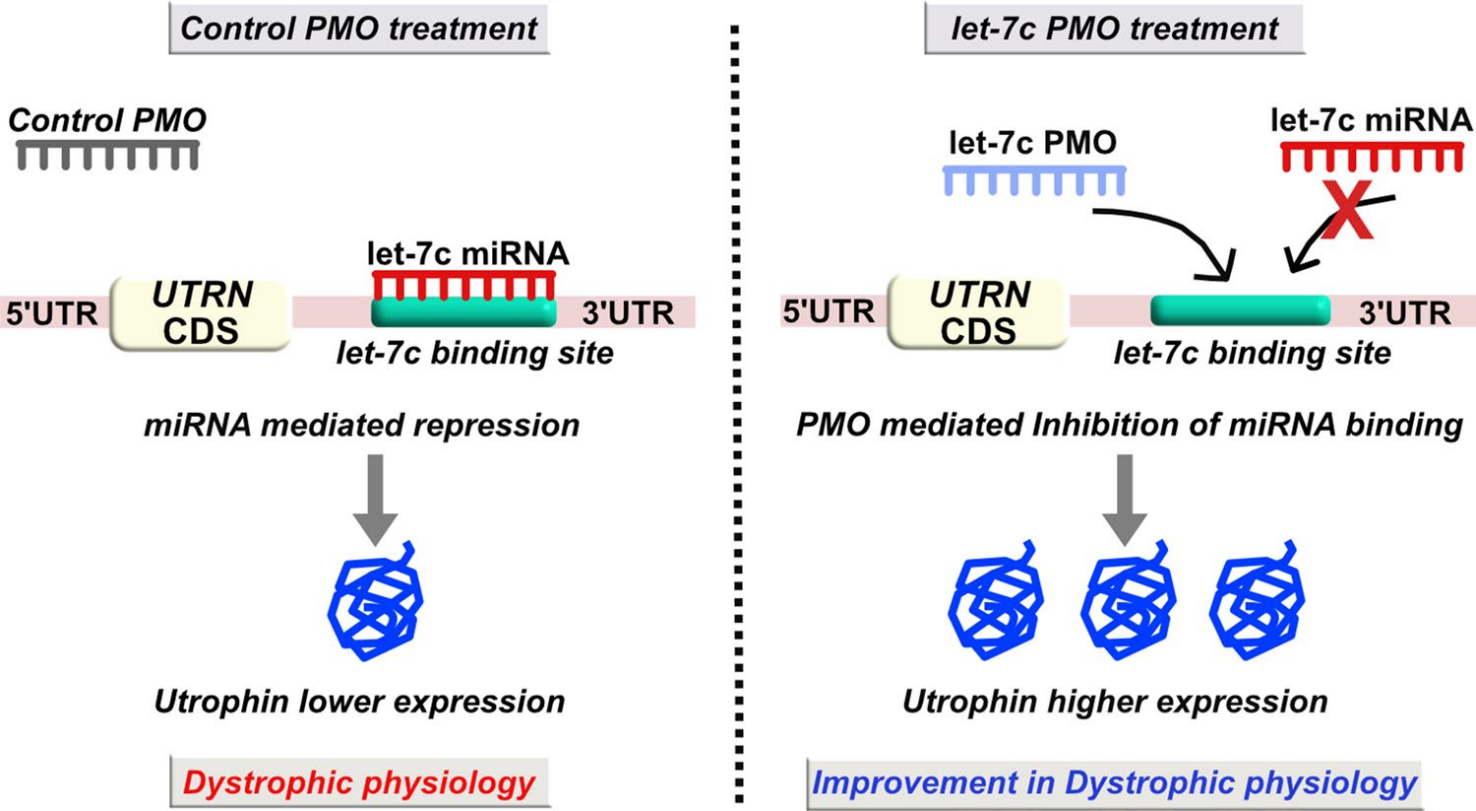
Schematic representation of the PMO-based let-7c SBO strategy to alleviate let-7c miRNA repression of utrophin gene. The left panel shows, let-7c miRNA mediated post-transcriptional repression of utrophin in control PMO treated mice. Whereas, the right panel shows let-7c PMO mediated blocking of miRNA binding to *UTRN* 3′UTR, resulting in higher utrophin expression and improvement in dystrophic pathophysiology.

In this study, we have designed five let-7c PMO-based SBOs (~ 24 to 29 bp) targeting different overlapping regions of the let-7c target site in the *UTRN* 3′UTR, in order to develop additional utrophin upregulation-based therapeutic strategies. These let-7c PMO-based SBOs differ in sequence and chemical composition compared to the 2OMePS-based SBOs we described previously. We used a variety of biochemical, molecular, and morphological methods to rank order and test their ability to upregulate utrophin and ameliorate the dystrophic phenotype in *mdx* mice. We propose that let-7c PMO-based SBOs are a promising utrophin-mediated therapeutic approach for DMD (Fig. 1).

## Results

### Let-7c PMO SBO treatment showed increased utrophin expression in C2C12 cells

**Figure 2:**
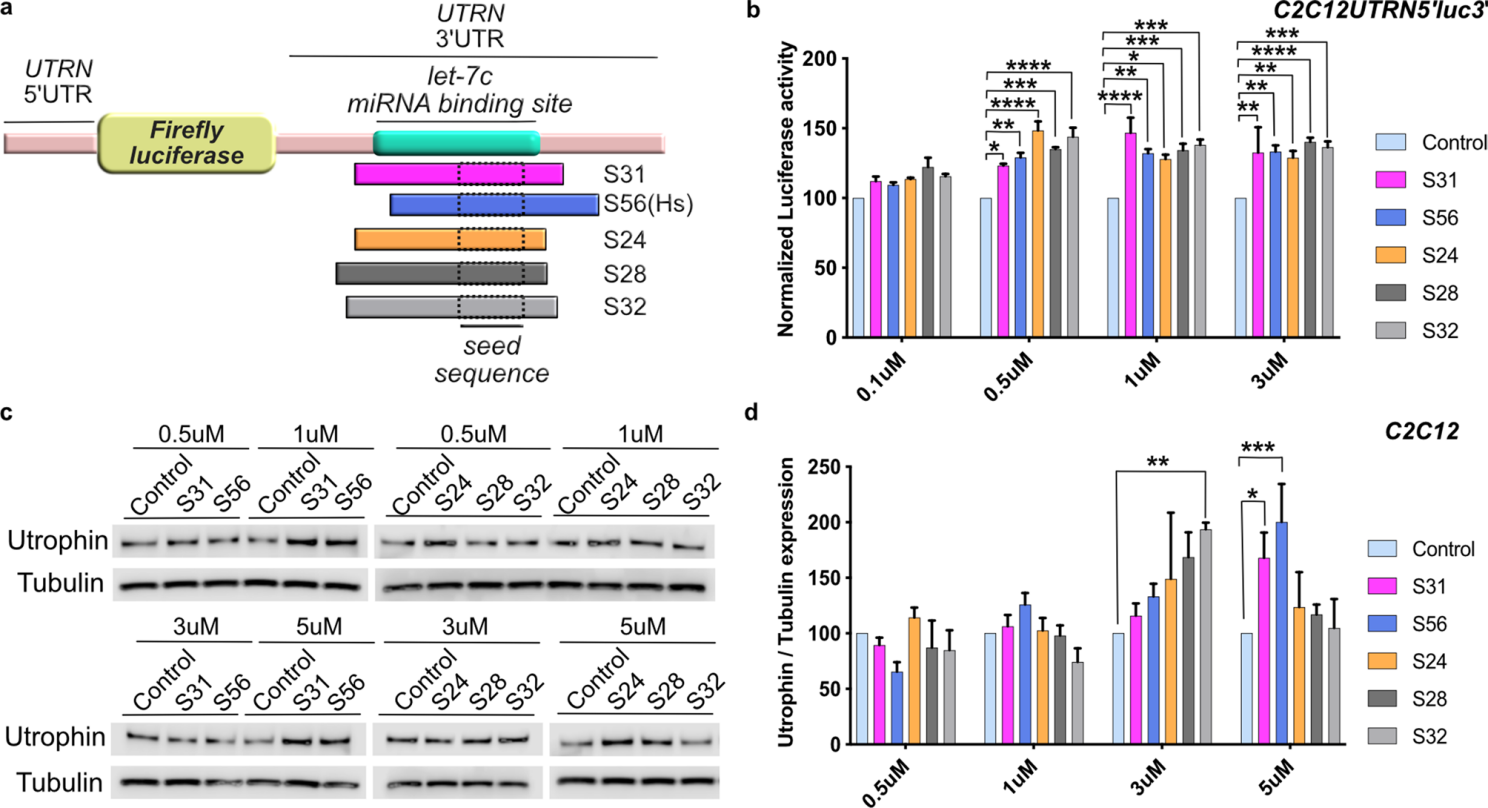
Let-7c PMO SBO upregulates utrophin expression in the C2C12 cell line. (**a**) Schematic diagram showing the relative position of the let-7c binding site (green) of five different let-7c PMO SBOs in *UTRN* 3′UTR. (**b**) Luciferase expression in C2C12*UTRN*5′luc3′ reporter cell line after treatment with 0.1 µM, 0.5 µM, 1 µM and 3 µM of control or five different let-7c PMO SBOs for 24 hrs. Data shown as percentage of normalized luciferase expression compared with control PMO treated cells (n = 3). (**c**) Endogenous utrophin expression in C2C12 cell line after treatment with 0.5 µM, 1 µM, 3 µM and 5 µM of control or five different let-7c PMO SBOs for 48 hrs. (**d**) Quantification of endogenous utrophin expression in C2C12 cell line shown as percentage of normalized utrophin expression compared with control PMO treated cells (n = 3). Each bar represents mean ± SEM. Statistical analysis performed by Two-way ANOVA Tukey’s multiple comparisons test *p < 0.1, **p < 0.01, ***p < 0.001, ****p < 0.0001.

To determine the most efficient SBOs for achieving utrophin upregulation, we designed five different let-7c PMO SBOs (S24, S28, S31, S32 and S56) spanning different regions of the let-7c target site in the *UTRN* 3′UTR (Fig. 2a, Supplementary Table 1). To evaluate the efficacy of these PMOs, we used the C2C12*UTRN*5′luc3′ reporter cell line^39^ containing the luciferase gene flanked by *UTRN* 5′ and 3′ UTRs for reporter assays. Four different concentrations for each PMO, were tested in the reporter cell line. S24 and S31 PMO treated cells showed ~ 1.5-fold higher luciferase activity compared to control PMO treated cells at 0.5 µM and 1 µM concentration respectively (Fig. 2b). The higher activity of S24 and S31 PMO was noted in a narrow range of concentrations. However, S56 PMO, which was designed based on the human *UTRN* let-7c sequence, showed ~ 1.3-fold increase in luciferase activity from 0.5 µM to 3 µM concentration, implying effectiveness over a wider range of active concentrations. Also the S32 PMO showed ~ 1.4-fold increase in luciferase activity from 0.5 µM to 3 µM concentration (Fig. 2b, Supplementary Table 3).

We further tested if the five PMOs could upregulate endogenous utrophin protein levels in C2C12 cells by using western blotting as an independent, orthogonal assay. Consistent with the results obtained with the reporter cell line, S56 PMO showed the highest level of utrophin expression (~ two-fold upregulation) compared to cells treated with control PMOs. Whereas the S32 PMO showed ~ 1.9-fold higher utrophin expression at 3 µM but not at other concentrations (Fig. 2c,d, Supplementary Table 4). Based on these findings S56 PMO was chosen for an in vivo preclinical study in the *mdx* mouse model of DMD. *Mdx* mice have a premature stop codon mutation in exon 23 of the *DMD* gene, and are widely used for preclinical animal studies for DMD^40,41^.

### Systemic administration of S56 PMO upregulates utrophin in *mdx* mice

**Figure 3:**
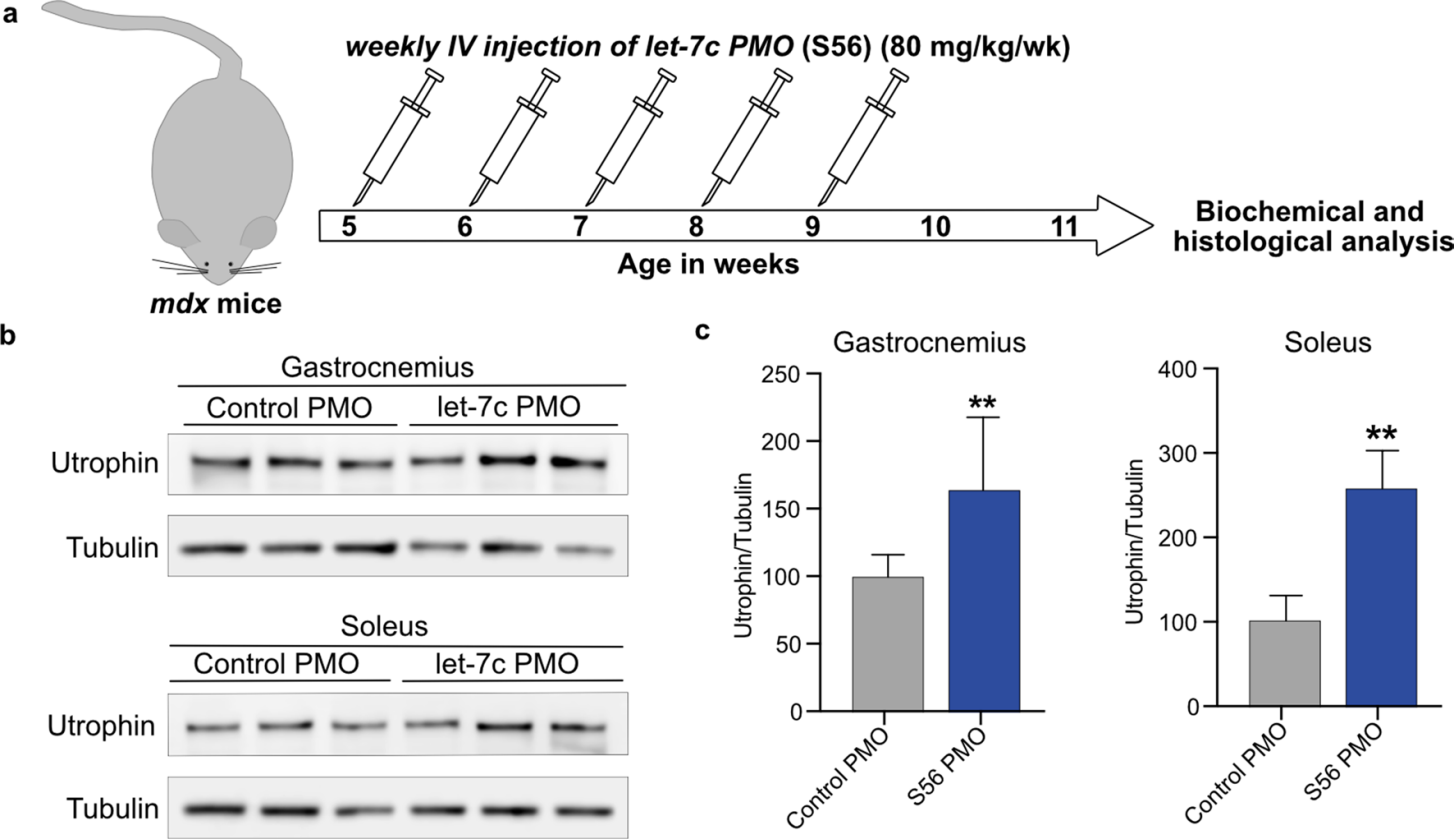
Higher expression of utrophin in *mdx* mice after five weeks of treatment with 80 mg/kg/wk S56 PMO. (**a**) Diagram of the experimental pipeline adopted for the study. (**b**) Western blot showed higher level of utrophin expression in gastrocnemius and soleus muscles of S56 PMO treated *mdx* mice. Samples from three different *mdx* mice treated with control or S56 PMO were shown. (**c**) Quantification of normalized utrophin level in control and S56 PMO treated *mdx* mice gastrocnemius and soleus muscle (n = 6 mice for both groups). Data shown as percentage of normalized utrophin level compared to control PMO treated *mdx* mice. Each bar represents mean ± SEM. Statistical analysis performed by Mann–Whitney nonparametric test, **p < 0.01.

PK/pharmacodynamic (PD) studies of PMOs have shown highest bioavailability upon intravenous (i.v) administration^35^. We therefore treated five weeks old *mdx* mice with five weekly retro-orbital injections of S56 PMO or control PMO at a dosage of 80 mg/kg. Two weeks after the end of the treatment, mice were sacrificed, and their skeletal muscles were examined (Fig. 3a). Western blots showed significant increases in utrophin protein expression in gastrocnemius (~ 1.6-fold) and soleus (~ 2.5-fold) muscles treated with S56 PMO compared to control PMO treated mice (Fig. 3b,c). Surveyed muscles from S56 and control PMO treated mice showed higher utrophin protein and mRNA expression levels, consistent with the intervention (Supplementary Figs. 1–2).

We further tested for expression of β-dystroglycan, which is a hallmark for restoration of dystrophin-glycoprotein complex (DGC). Western blots showed partial restoration of β-DG in S56 PMO treated TA and EDL muscles (Supplementary Fig. 3).

### S56 PMO treatment increased sarcolemmal utrophin expression in *mdx* mice

**Figure 4:**
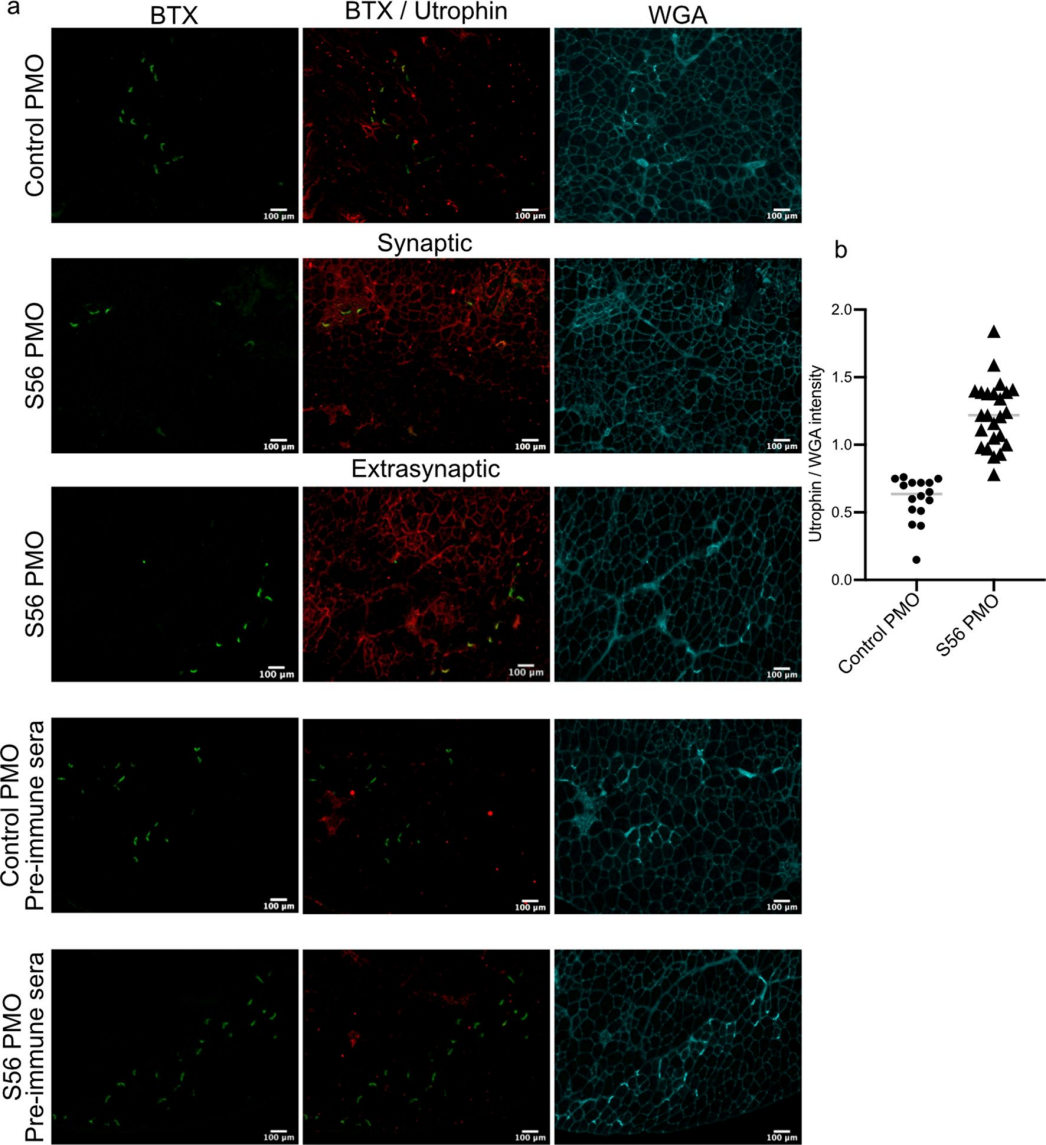
Immunohistochemistry of *mdx* TA muscles treated with S56 PMO showed higher sarcolemmal expression of utrophin. (**a**) TA muscles were stained with α-bungarotoxin (green), utrophin antibody (red) and wheat germ agglutinin (cyan). The control PMO and S56 PMO treated cryosections were also stained with utrophin pre-immune sera as control. Scale bar = 100 µm. The figures showed synaptic expression of utrophin in control PMO, and both synaptic and extra-synaptic sarcolemma associated expression of utrophin in S56 PMO treated mice. (**b**) Quantification of utrophin expression in sarcolemma normalized with WGA expression. S56 PMO treated muscles showed significantly higher expression of utrophin (****p = 0.0001, n = 4 mice for both groups). Statistical analysis performed by Mann–Whitney nonparametric test.

As the western blots showed higher expression of utrophin protein in S56 PMO treated *mdx* mice, we examined utrophin protein expression in the sarcolemma of control and S56 PMO treated *mdx* mice by immunohistochemistry. Control PMO treated TA sections showed utrophin expression primarily restricted to the neuromuscular junctions, as indicated by the colocalization with the synaptic marker α-Bungarotoxin (BTX). On the other hand, muscles from mice treated with S56 PMO showed increased expression of utrophin across both synaptic and extrasynaptic regions (Fig. 4a). Quantification of utrophin level normalized to wheat germ agglutinin (WGA) levels in sarcolemma showed ~ two-fold higher expression of utrophin in TA muscles of S56 PMO treated *mdx* mice (Fig. 4b).

### S56 PMO treated *mdx* mice showed decrease in muscle degeneration and morphological improvement

**Figure 5:**
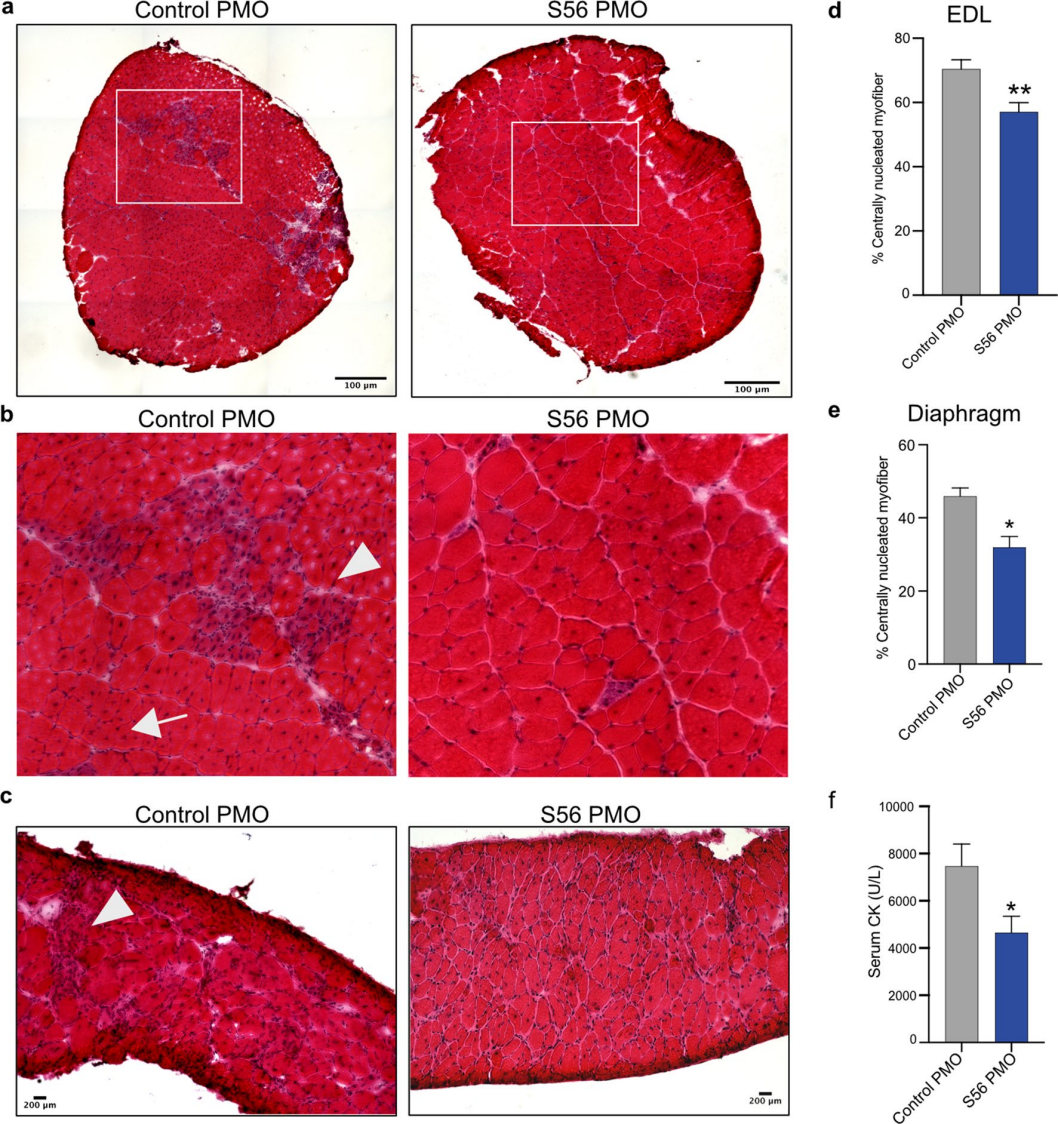
H&E staining of cryosections from control PMO or S56 PMO treated *mdx* mice EDL and diaphragm showed morphological improvement. (**a**) Representative H&E staining image of whole EDL cryosections (scale bar = 100 µm). (**b**) Regions highlighted by the white boxes were magnified (×3) (scale bar = 25 μm). (**c**) Representative H&E staining of diaphragm cryosections (scale bar = 200 µm). Control PMO treated EDL and diaphragm muscle cryosections show regenerated myofibers (arrow) and immune cell infiltration (arrowhead) and S56 PMO treatment appeared to alleviate symptoms. (**d, e**) Quantification of centrally nucleated EDL and diaphragm muscle fibers of *mdx* mice treated with control PMO or S56 PMO. The graphs show significant decrease in the percentage of CNFs of EDL and diaphragm muscles in S56 PMO treated *mdx* mice (**p = 0.002, n = 10 mice for both groups of EDL muscles, *p = 0.0140, n = 6 mice for both groups of diaphragm muscles). (**f**) The graph shows serum CK levels in S56 PMO treated mice were significantly lower than control PMO treated *mdx* mice (*p = 0.02, n = 10 mice for both groups). Each bar represents mean ± SEM. Statistical analysis performed by Mann–Whitney nonparametric test.

We further examined whether the dystrophic histopathology was improved by S56 PMO treatment compared to control PMO treatment in *mdx* mice. H&E staining of EDL and diaphragm sections of control PMO treated *mdx* mice showed regenerating centrally nucleated fibers (CNFs) and immune cell infiltration as previously reported^40^. However, let-7c PMO treatment of age-matched *mdx* mice showed improvement in the pathophysiology, with reduced CNFs, necrosis and cellular infiltration observable in EDL and diaphragm (Fig. 5a–c). The higher percentage of CNFs is one of the well-recognized, characterized and objectively scored morphological characteristics of dystrophic muscles, attributed to continuous muscle regeneration^40,43^. Two weeks after the end of treatment, EDL and diaphragm muscles of *mdx* mice showed significantly lower percentage of CNFs in S56 PMO treated mice (c.a. 13% reduction) compared to control PMO treated mice, providing morphological evidence of improvement of the dystrophic phenotype (Fig. 5d,e). The morphological improvements noted in terms of reduction in the percentage of CNFs and muscle degeneration were not accompanied by changes in fiber size or contractile properties (Supplementary Table 2).

### S56 PMO treatment decreases serum CK levels in *mdx* mice

We further studied the therapeutic effects of the let-7c PMO treatment on *mdx* mice by examining the levels of serum CK. Elevated CK level is one of the hallmarks of dystrophic pathophysiology in mice and humans^40,42^. Two weeks after the end of the treatment, serum CK was significantly lower (c.a. 38% reduction) in S56 PMO treated compared to control PMO treated *mdx* mice (Fig. 5f). The decrease in serum CK provides biochemical evidence of utrophin mediated improvement in dystrophic pathology.

## Discussion

In this study, we developed and used the S56 PMO-based let-7c SBO to alleviate miRNA mediated repression of utrophin expression and achieved improvement in *mdx* pathophysiology i*n vivo*. Utrophin mRNA translation is known to be inhibited by a set of six miRNAs^25,26^. Bulaklak et al., showed AAV mediated expression of a miR-206 sponge decoy^44,45^ in *mdx* mice, resulted in utrophin upregulation and improvement in the dystrophic phenotype, independently demonstrating the importance of miRNA mediated regulation of utrophin expression^27^. We had shown higher utrophin expression and improvement of the dystrophic phenotype by blocking the let-7c target site in *UTRN* 3′UTR using a 2OMePS-based let-7c SBO *in vivo*^30^. Additionally, sPIF-mediated let-7 downregulation has also been shown to result in utrophin upregulation and improvement in the dystrophic phenotype^46^, underscoring the significance of let-7 mediated regulation of utrophin expression. However, the clinical application of 2OMePS-based let-7c SBO for utrophin upregulation or 2OMePS-based *DMD* exon skipping^47^ AONs is somewhat compromised by their suboptimal PKs and therapeutic efficacy^34^. Whereas, PMOs while clinically approved by the FDA, have sub-optimal efficacy^36,37,48^. PK studies of PMOs show rapid elimination from bloodstream and limited entry to mature muscle fibers^49^. However, a recent study by Novak *et. al.* demonstrated presence of *DMD* exon skipping PMOs in macrophages at the site of myofiber lesions for several days after elimination of PMOs from bloodstream, demonstrating the availability of PMOs at actively regenerating myofibers^50,51^. Here, we tested the efficacy of five different let-7c PMO-based SBOs for utrophin upregulation in vitro and studied the most efficient PMO (S56 PMO) in *mdx* mice to demonstrate improvement in dystrophic pathophysiology.

AAV mediated μ-utrophin delivery^52–54^ and artificial transcriptional factor mediated transcriptional upregulation of full length utrophin^24,55^ respectively, have been shown to improve dystrophic pathophysiology in the *mdx* model. Notably, μ-utrophin expression does not induce significant immune responses compared to μ-dystrophin expression in deletional animal models of DMD^54^. In this study, weekly S56 PMO administration for 5 weeks resulted in ~ 2.5-fold and ~ 1.6-fold higher level of utrophin in soleus and gastrocnemius muscle respectively, biochemical and morphological improvement as demonstrated by a 38% reduction in serum CK levels and ~ 13% reduction in percentage of CNFs compared to control PMO treated *mdx* mice (Figs. 3, 4, 5). While improvement in these parameters are extremely encouraging, the improvement achieved at this dose was limited (Supplementary Table 2). Our previous studies using two different doses (10 mg/kg and 100 mg/kg) of 2OMePS let-7c SBO showed dose-dependent utrophin protein upregulation in skeletal and diaphragm muscle and improvement in mechanical properties only at the higher dose^30^. However, even at the higher dose of 2OMePS let-7c SBO, the serum CK level showed no improvement. Here, with a relatively low dose of 80 mg/kg S56 PMO treatment for five weeks in *mdx* mice showed increased expression of utrophin, morphological improvement and a decrease in serum CK level. We were unable to test higher doses of our lead PMO-based let-7c SBO due to volume constraints of retrobulbar delivery, coupled with the high GC content of the target site, which limited the solubility of the S56 PMO SBO we developed. We believe that designing and testing of additional SBOs and/or use of alternate chemistries (e.g. Pep-PMO, FANA) would help overcome this limitation^56,57^, and we are currently undertaking these experiments to increase the potential therapeutic efficacy.

We suggest that the functional improvement we achieved can be further improved with an earlier onset of treatment, a longer period of treatment and/or administration of higher PMO dosages. Given that PMOs have demonstrated sufficient safety and efficacy in vivo at higher doses (100–300 mg/kg/wk)^48,58–60^, the 80 mg/kg/wk we used should not be limiting, however, additional sequences based on S56 would need to tested to quantify dose-dependent improvements. Additionally, here we have targeted one specific miRNA::utrophin interaction, however this approach can easily be extended to additional miRNAs that we and others have identified^25,26^ singly and in combination, to extend the potential benefits. Based on these findings, we propose that PMO-based utrophin miRNA SBOs are a promising approach for achieving utrophin mediated therapeutics for DMD.

## Methods

### Cell culture

C2C12 mouse myoblast cell lines (both from ATCC) and the C2C12*UTRN*5′luc3′ stable cell lines^39,61^ were maintained in standard growth condition in DMEM high glucose (Gibco, MD) supplemented with 10% fetal bovine serum (Sigma-Aldrich, MO) and 1% Pen/Strep (Gibco, MD).

### PMO-based SBO design

The PMO backbone let-7c SBOs were designed to specifically target and block the let-7c miRNA target site in *UTRN* 3′UTR. Five let-7c PMOs were designed targeting the same let-7c site but with different flanking regions considering the binding efficiency. A control PMO was designed with the scramble sequence. All the PMOs were synthesized by Gene Tools, LLC (OR) (Supplementary Table 1).

### Luciferase assay

The C2C12*UTRN*5′luc3′ cell line was seeded at 50,000 cells/well in 24 well plates and allowed to attach for O/N as described^39^. Next day, cells were treated for 24hrs. with control or let-7c PMOs of desired concentrations (0.1 µM, 0.5 µM, 1 µM, 3 µM) along with 6 µM transfection reagent endoporter (Gene Tools, LLC, OR). The next day, treated cells were lysed using the passive lysis buffer (Promega, WI) supplemented with complete protease inhibitor (Roche, Switzerland) and total protein contents were measured with the Pierce BCA protein assay kit (Thermo Fisher Scientific, MA). Luciferase assays were done using the luciferase assay system (Promega, WI) in a 96 well plate and quantified using a Cytation5 plate reader (BioTek, VT). Luciferase activity was normalized to the total protein loaded.

### Animal studies

All animal procedures were reviewed and approved by the Institutional Animal Care and Use Committee at the University of Pennsylvania (UPenn). The C57BL/10ScSn-*mdx*/J mouse model of DMD (*mdx*) was utilized for all experiments. Breeding pairs were obtained from the Jackson Laboratory (Bar Harbor, ME). Mice were housed and bred at the UPenn animal facility, provided food, water ad libitum, and maintained under 12 h. light/dark cycles.

### Preclinical studies in *mdx* mice with PMOs

PMOs were solubilized in saline and warmed at 50 °C for 15mins and cooled down to room temperature before injections. Five-week-old male *mdx* mice were anesthetized with 4% isoflurane and control or S56 PMO was administered systemically via the retro-orbital sinus as a single 80 mg/kg dose (< 250 µl volume) using a 28-gauge/0.5 ml insulin syringe, weekly for 5 weeks. 2 weeks after the last injection, mice were anesthetized by 4% isoflurane and sacrificed. E*x vivo* contractility analyses were performed as previously described^30^. Following the procedure, muscles were flash-frozen in liquid nitrogen-cooled isopentane and stored at − 80 °C.

### Tissue harvesting and cryosectioning

Muscle samples were surgically removed after euthanasia, embedded in OCT, flash frozen in liquid nitrogen-chilled isopentane and stored at − 80 °C. For immunohistochemistry and H&E staining, tissues were sectioned at 5 µm thickness on a Leica CM1950 cryostat and stored at − 80 °C.

### Immunohistochemistry

Cryosections were blocked for 1 h. with 4% BSA in PBS, followed by staining with custom made rabbit anti-utrophin A antibody raised with the N-terminal utrophin A peptide (CMAKYGDLEARPDDGQNE) as described before^62^ (1:500, ProSci Inc, CA) overnight at 4 °C in a humidified chamber. Sections were then washed and incubated with the goat anti-rabbit Alexa Fluor 488 conjugated secondary antibody (Life Technologies, MA) at a dilution of 1:400 for 1 h. at room temperature. α-Bungarotoxin and Wheat germ agglutinin (WGA) conjugated to Alexa Fluor 594 or 647 (Life Technologies, MA) were used at a dilution of 1:400 for 30 mins at room temperature. Sections were mounted with Prolong Gold Antifade Mounting Media with DAPI (Thermo Fisher Scientific, MA) for nuclear staining.

### Western blots

Frozen tissue samples were cut with pre-cooled scissors and minced in a tube pre-cooled in dry ice. The samples were then suspended in muscle denaturing buffer (50 mM Tris–HCl pH 7.5, 150 mM NaCl, 1% Triton X100, 1 mM EDTA, 10 mM MgCl_2_, 1% SDS, 0.5 mM DTT, 10% glycerol) with complete protease inhibitor cocktail (Roche, Basel, Switzerland). The equilibrated tissue samples were homogenized using a TissueLyser II (Qiagen, Hilden, Germany) with 5 mm stainless steel beads (Thermo Fisher Scientific, MA) at a frequency of 20 Hz for 2 min. C2C12 cells were lysed with RIPA buffer (Thermo Fisher Scientific, MA) with protease inhibitor cocktail. Total protein was measured by Pierce BCA Protein assay kit (Thermo Fisher Scientific, MA). 10 µg of total protein was resolved in 3–8% Tris–Acetate protein gel (NuPAGE, Thermo Fisher Scientific, MA) and transferred to a nitrocellulose membrane using Trans-Blot Turbo Transfer System (Bio-Rad, CA). For western blotting, membranes were first blocked with 5% non-fat dry milk in TBS with 1% Tween 20 for 1 h. at room temperature. After blocking, blots were incubated with the following primary antibodies; mouse monoclonal anti-utrophin (1:100, Mancho3(8A4), developed by Prof. Glenn E. Morris; DSHB, Iowa) and mouse anti-α-Tubulin (1:5000, T6199, Sigma-Aldrich, MO) overnight at 4 °C. Next day, blots were washed; incubated with mouse IgGκ binding protein (m-IgGκ BP) conjugated to horseradish peroxidase (HRP) (1:2500, sc-516102, Santa Cruz Biotechnology, TX); washed and developed using Immobilon Western Chemiluminescent HRP Substrate (MilliporeSigma, MA) and imaged in LI-COR C-Digit Blot Scanner (LI-COR Biosciences, NE).

### Serum CK assay

Blood samples were collected via cardiac puncture under deep terminal anesthesia, centrifuged at 2000 g for 5 min and serum stored at − 80 °C until analysis. CK levels were determined by the Clinical Pathology Laboratory at the Matthew J. Ryan Veterinary Hospital of the University of Pennsylvania.

### Statistical analysis

Data were analyzed using the GraphPad Prism v8 statistical software package. Values are presented as mean ± standard error of the mean (SEM). Statistical analysis was performed using Mann–Whitney test or Two-Way ANOVA multiple comparisons with statistical significance level set at α ≤ 0.05.

### Statement of Ethical Approval

All methods performed in this study were in accordance with relevant guidelines, regulations and with full approval.

## Supplementary information

Supplementary Information.
