# Supplementary material for: PMO-based let-7c site blocking oligonucleotide (SBO) mediated utrophin upregulation in mdx mice, a therapeutic approach for Duchenne muscular dystrophy (DMD)
Source: Sci Rep. 2020 Dec 9;10:21492. doi: 10.1038/s41598-020-76338-1 (PMC7726560; doi:10.1038/s41598-020-76338-1)

**Supplementary Information**

**PMO-based let-7c site blocking oligonucleotide (SBO) mediated utrophin upregulation in *mdx* mice, a therapeutic approach for Duchenne Muscular Dystrophy (DMD)**

Kasturi Sengupta^1^, Emanuele Loro^1^ & Tejvir S. Khurana^1^

^1^Department of Physiology and Pennsylvania Muscle Institute, Perelman School of Medicine, University of Pennsylvania, Philadelphia, PA 19104, USA.

***Correspondence:** Tejvir S. Khurana, Department of Physiology and Pennsylvania Muscle Institute, Perelman School of Medicine, University of Pennsylvania, 755 Clinical Research Building, Philadelphia, PA 19104, USA.

**E-mail:** tsk@pennmedicine.upenn.edu

**Supplementary Table 1. PMO sequences**

| PMO Oligo | Sequences 5’-3’ | Length (mer) |
| --- | --- | --- |
| S24 | CTGAGGTAGAAAGGTGATCATGGCTC | 26 |
| S28 | CTGAGGTAGAAAGGTGATCATGGCTCTCC | 29 |
| S31 | GTTCTGAGGTAGAAAGGTGATCATGGCTC | 29 |
| S32 | TCTGAGGTAGAAAGGTGATCATGGCTCT | 28 |
| S56 | AAGATGGATCTGAGGTAGAAAGGT | 24 |
| Control | GTGAGCACTTCTTTCCTTCTTTTTT | 25 |

**Supplementary Table 2. Comparison of morphological and physiological properties of EDL muscle (n=10)**

|  | 80 mg/kg/wk | | *P* value |
| --- | --- | --- | --- |
|  | Control PMO | S56 PMO |  |
| Weight (mg) | 13.3±0.48 | 12.1±0.37 | 0.06 |
| Cross sectional area (CSA) (mm^2^) | 2.36±0.07 | 2.17±0.06 | 0.03^*^ |
| Absolute Tetanic force (mN) | 379.72±17.7 | 362.15±15.6 | 0.38 |
| Specific Tetanic force (N/cm^2^) | 16.10±0.62 | 16.7±0.57 | 0.86 |
| ECC force decrease (1-4) (%) | 77.26±1.92 | 72.93±1.86 | 0.06 |
| ECC force drop (4^th^) (%) | 22.74±1.92 | 27.07±1.86 | 0.06 |
| Avg. of Minimal Feret’s diameter (µm) | 31.58±0.7 | 36.37±2.29 | 0.4 |
| Variance coefficient of Min. Feret’s diameter | 305.27±14.5 | 321±16.33 | 0.7 |

Results are presented as mean ± SEM; S56 PMO treated group were compared with control PMO treated group and statistical significance were analyzed by Mann-Whitney test.

**Supplementary Table 3. Statistical significance data of luciferase expression in C2C125’*UTRN*3’ cells treated with control and let-7c PMO**

| Tukey’s Multiple comparisons test | significance | Adjusted P value |
| --- | --- | --- |
| 0.1 µM | | |
| Control vs. S31 | ns | \| 0.6332 \| \| --- \| |
| Control vs. S56 | ns | 0.8349 |
| Control vs. S24 | ns | 0.5218 |
| Control vs. S28 | ns | \| 0.0697 \| \| --- \| |
| Control vs. S32 | ns | \| 0.3654 \| \| --- \| |
| 0.5 µM | | |
| Control vs. S31 | \| * \| \| --- \| | \| 0.0495 \| \| --- \| |
| Control vs. S56 | \| ** \| \| --- \| | 0.0068 |
| Control vs. S24 | \| **** \| \| --- \| | <0.0001 |
| Control vs. S28 | \| *** \| \| --- \| | 0.0006 |
| Control vs. S32 | \| **** \| \| --- \| | <0.0001 |
| 1 µM | | |
| Control vs. S31 | \| **** \| \| --- \| | <0.0001 |
| Control vs. S56 | \| ** \| \| --- \| | \| 0.0021 \| \| --- \| |
| Control vs. S24 | \| * \| \| --- \| | \| 0.0103 \| \| --- \| |
| Control vs. S28 | \| *** \| \| --- \| | \| 0.0009 \| \| --- \| |
| Control vs. S32 | \| *** \|  \| \| --- \| --- \| | 0.0002 |
| 3 µM | | |
| Control vs. S31 | \| ** \|  \| \| --- \| --- \| | 0.0018 |
| Control vs. S56 | \| ** \|  \| \| --- \| --- \| | 0.0013 |
| Control vs. S24 | \| ** \|  \| \| --- \| --- \| | 0.0072 |
| Control vs. S28 | \| **** \|  \| \| --- \| --- \| | <0.0001 |
| Control vs. S32 | \| *** \|  \| \| --- \| --- \| | 0.0004 |

**Supplementary Table 4. Statistical significance data of utrophin expression in C2C12 cells treated with control and let-7c PMO**

| Tukey’s Multiple comparisons test | significance | Adjusted P value |
| --- | --- | --- |
| 0.5 µM | | |
| Control vs. S31 | ns | \| 0.9960 \| \| --- \| |
| Control vs. S56 | ns | 0.5696 |
| Control vs. S24 | ns | 0.9884 |
| Control vs. S28 | ns | 0.9928 |
| Control vs. S32 | ns | \| 0.9847 \| \| --- \| |
| 1 µM | | |
| Control vs. S31 | ns | 0.9997 |
| Control vs. S56 | ns | 0.8141 |
| Control vs. S24 | ns | >0.9999 |
| Control vs. S28 | ns | >0.9999 |
| Control vs. S32 | ns | 0.8614 |
| 3 µM | | |
| Control vs. S31 | ns | 0.9722 |
| Control vs. S56 | ns | 0.6111 |
| Control vs. S24 | ns | 0.4046 |
| Control vs. S28 | ns | 0.0947 |
| Control vs. S32 | ** | 0.0073 |
| 5 µM | | |
| Control vs. S31 | * | 0.0229 |
| Control vs. S56 | *** | 0.0002 |
| Control vs. S24 | ns | 0.9389 |
| Control vs. S28 | ns | 0.9853 |
| Control vs. S32 | ns | >0.9999 |

**Supplementary Figure legends**

**Supplementary Figure 1.** Utrophin protein and RNA expression in *mdx* mice treated with 80 mg/kg/week control PMO or S56 PMO for five weeks. Representative western blots show higher utrophin protein expression in S56 PMO treated TA (a), EDL (b) and diaphragm (c) tissues. Quantification of the normalized utrophin protein expression in control PMO and S56 PMO treated TA (d), EDL (e) and diaphragm (f) tissues. Data shown as percentage of normalized utrophin protein expression compared with control PMO treated mice. Each bar represents mean ± SEM (n=6). Statistical analysis performed by Mann-Whitney nonparametric test with statistical significance level set at α≤0.05. (**p*=0.04 for TA, **p*=0.03 for EDL and ***p*=0.003 for diaphragm). Utrophin RNA expression in the corresponding TA (g), EDL (h) and diaphragm (i) tissues. Data shown as fold change in normalized utrophin RNA expression compared with control PMO treated *mdx* mice tissues. Each bar represents mean ± SEM (n=4). Statistical analysis performed by Mann-Whitney nonparametric test with statistical significance level set at α≤0.05. (*p*=0.22 for TA, **p*=0.02 for EDL and *p*=0.2 for diaphragm).

**Supplementary Figure 2.** Utrophin RNA expression in the gastrocnemius and soleus muscles of *mdx* mice treated with 80 mg/kg/week control PMO or S56 PMO for five weeks. a-b. Data shown as fold changes in normalized utrophin RNA expression in S56 PMO treated *mdx* mice compared with control PMO treated *mdx* mice. Each bar represents mean ± SEM (n=4). Statistical analysis performed by Mann-Whitney nonparametric test with statistical significance level set at α≤0.05. (*p*=0.11 for gastrocnemius, **p*=0.02 for soleus).

**Supplementary Figure 3.** Restoration of β-DG in S56 PMO treated *mdx* mice. a & b. Representative western blots show expression of β-DG in the wild type mice and absence in *mdx* mice treated with control PMO. S56 PMO treated *mdx* mice show partial restoration of β-DG expression compared with control PMO treated *mdx* mice in TA and EDL tissues.

**Supplementary Methods**

**Total RNA isolation, reverse transcription and quantitative Real Time PCR (qPCR) from *mdx* mice tissue samples**

Frozen tissue samples were cut with pre-cooled scissors and minced in a tube pre-cooled in dry ice. The samples were then suspended with 500 µl of TRIzol reagent (Invitrogen) and homogenized using a Tissue Lyser II (Qiagen, Hilden, Germany) with 5 mm stainless steel beads (Thermo Fisher Scientific, MA) at a frequency of 20 Hz for 2 mins. Total RNA was harvested following manufacturers protocol. 1 µg of RNA from each sample were reverse transcribed with SuperScriptTM III Reverse Transcriptase (Thermo Fisher Scientific, MA) using random hexamer following manufacturers protocol. Quantitative PCR was performed in QuantStudio3 Real-Time PCR System (Applied Biosystems) using Power SYBR Green Master Mix (Applied Biosystem) as described before^1^.

**Supplementary Reference**

1 Mishra, M. K., Loro, E., Sengupta, K., Wilton, S. D. & Khurana, T. S. Functional improvement of dystrophic muscle by repression of utrophin: let-7c interaction. *PLoS One* **12**, e0182676, doi:10.1371/journal.pone.0182676 (2017).

Supplementary Figure 1


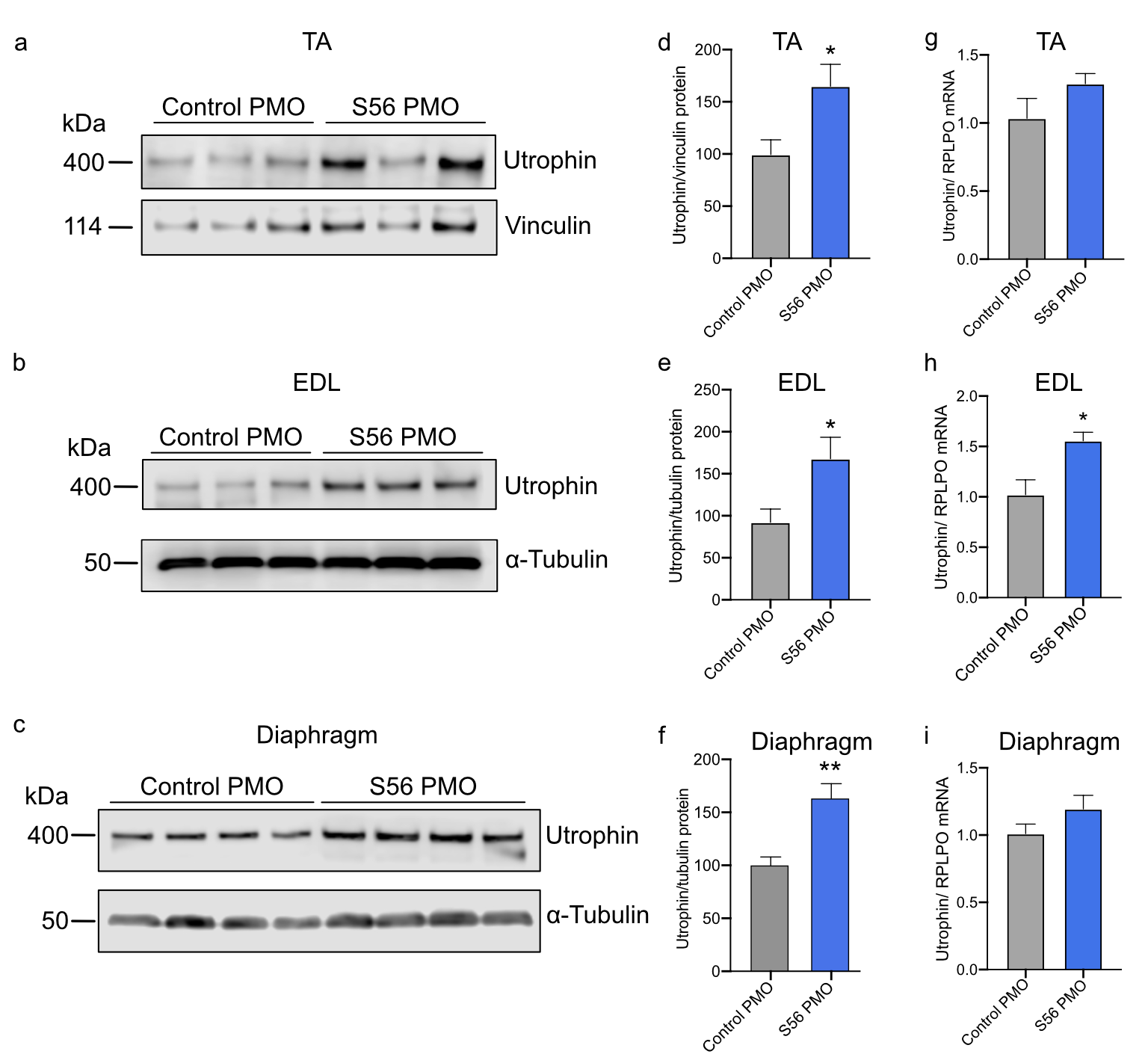


Supplementary Figure 2


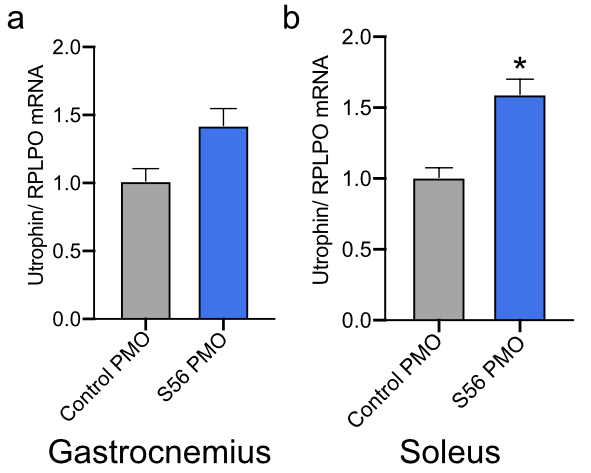


Supplementary Figure 3:


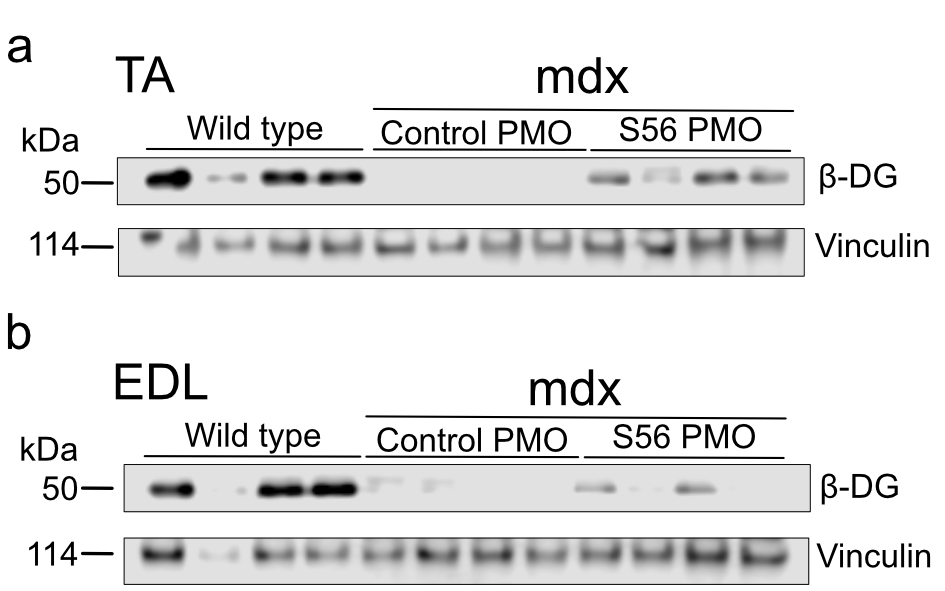


Western blot Images

Figure 2 c.


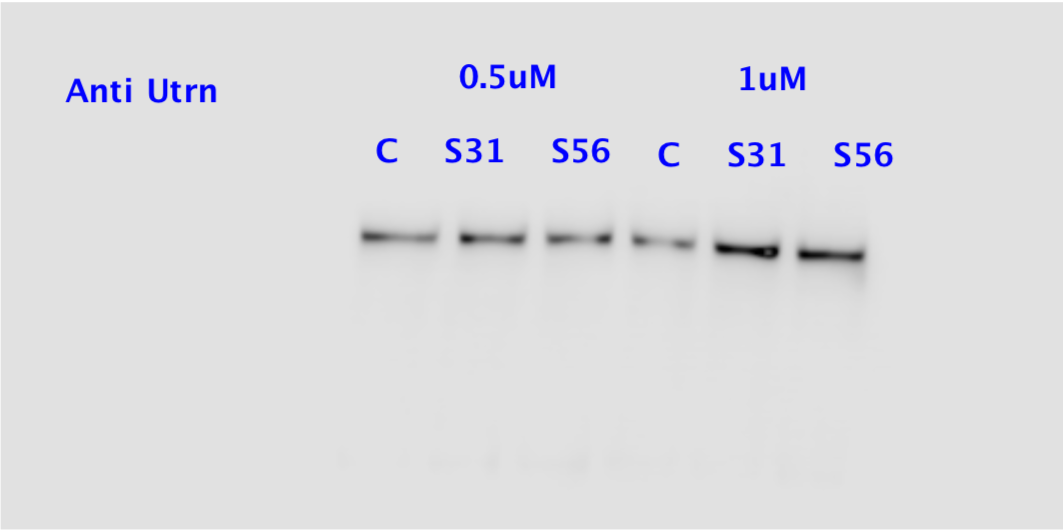


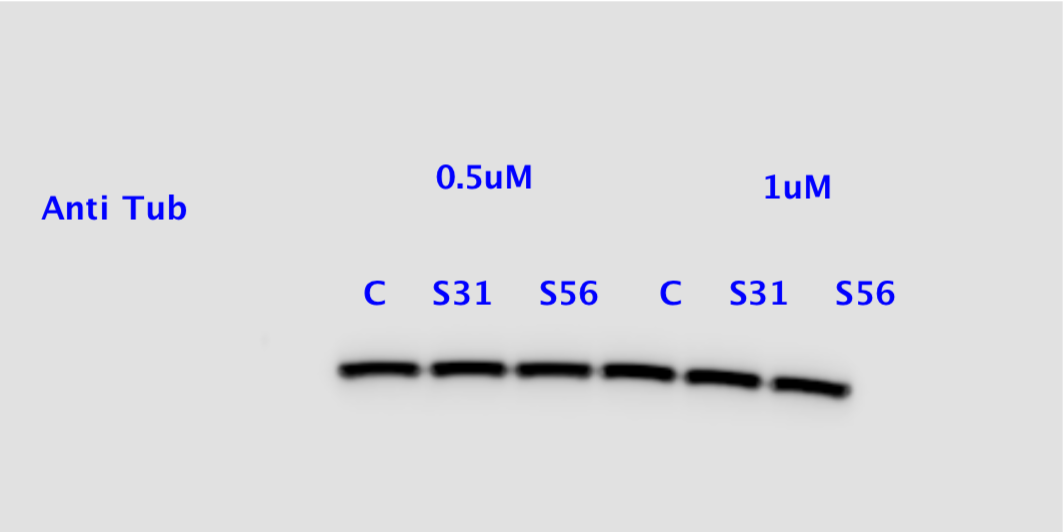


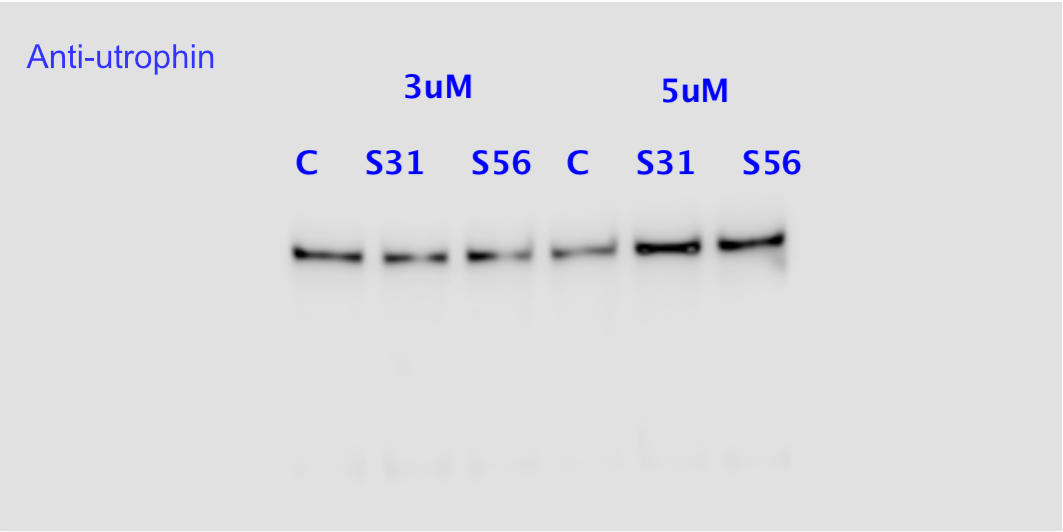


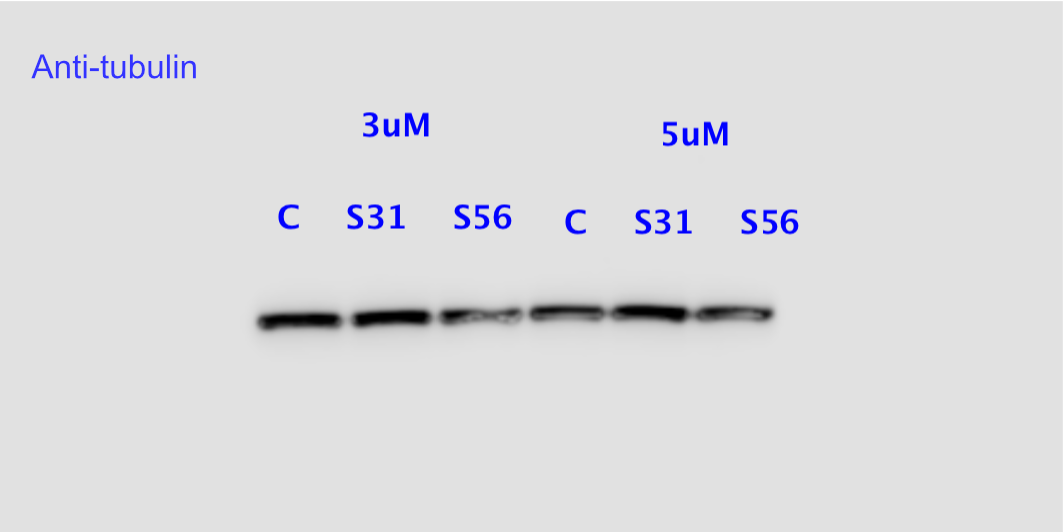


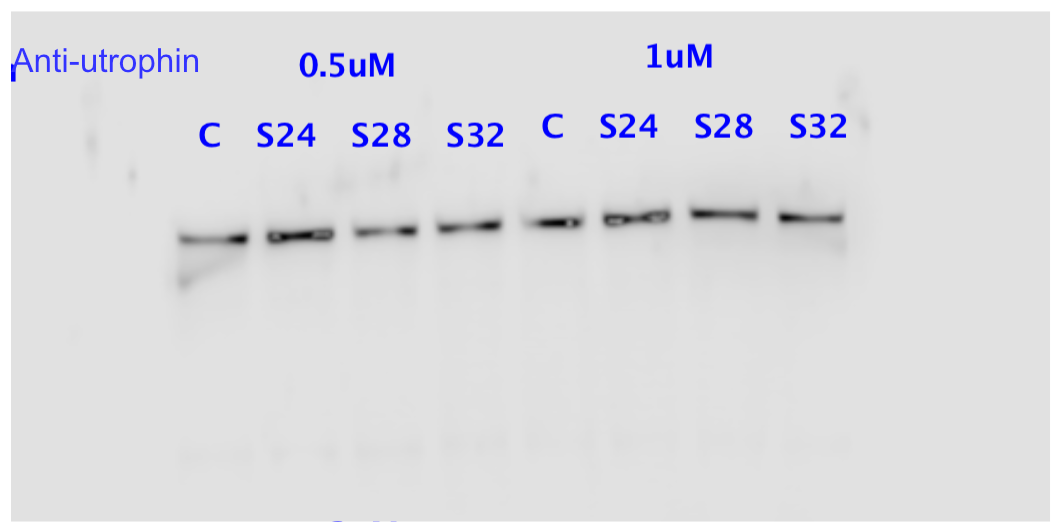


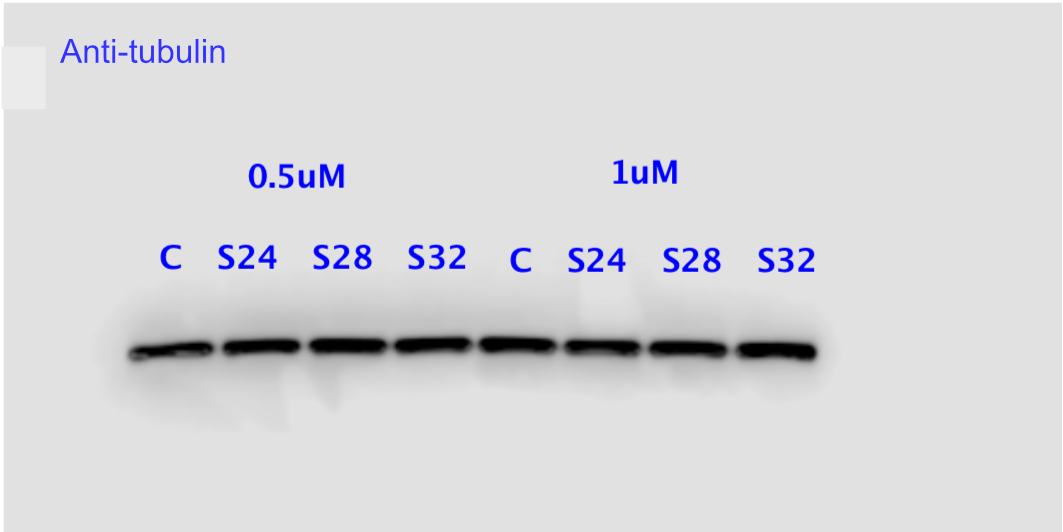


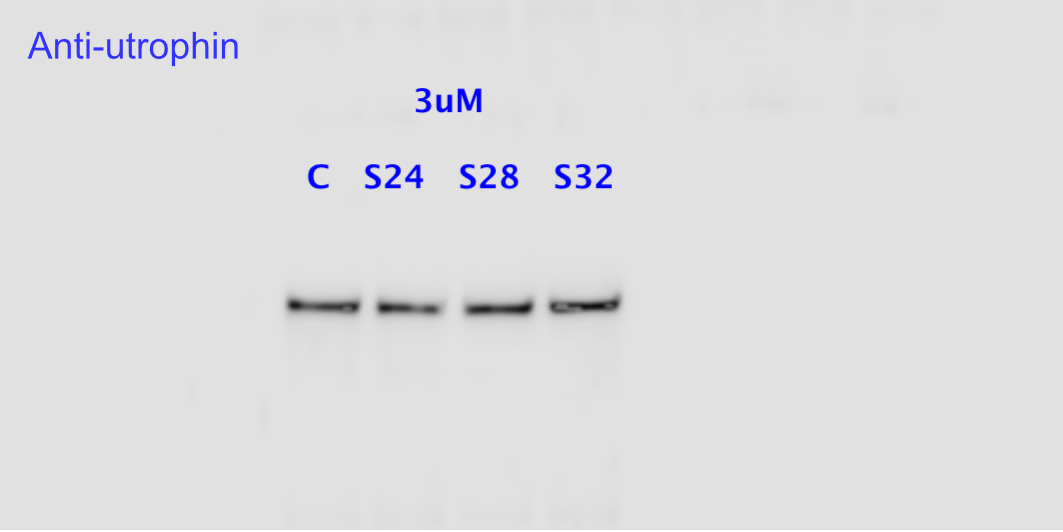


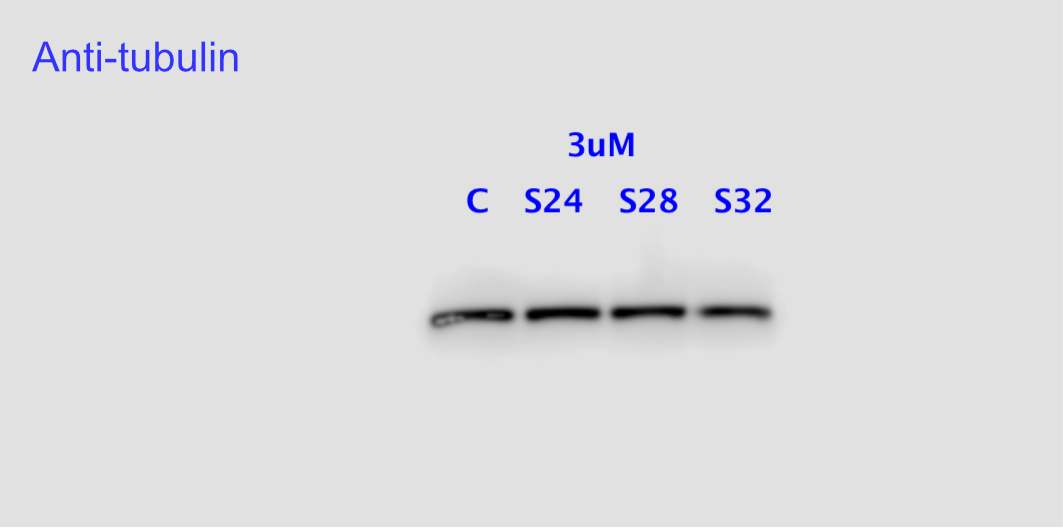


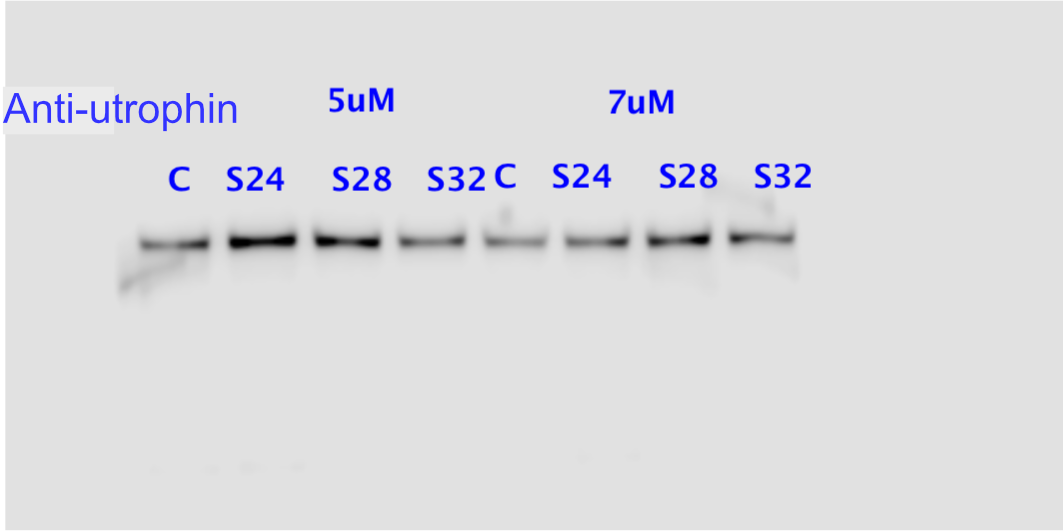


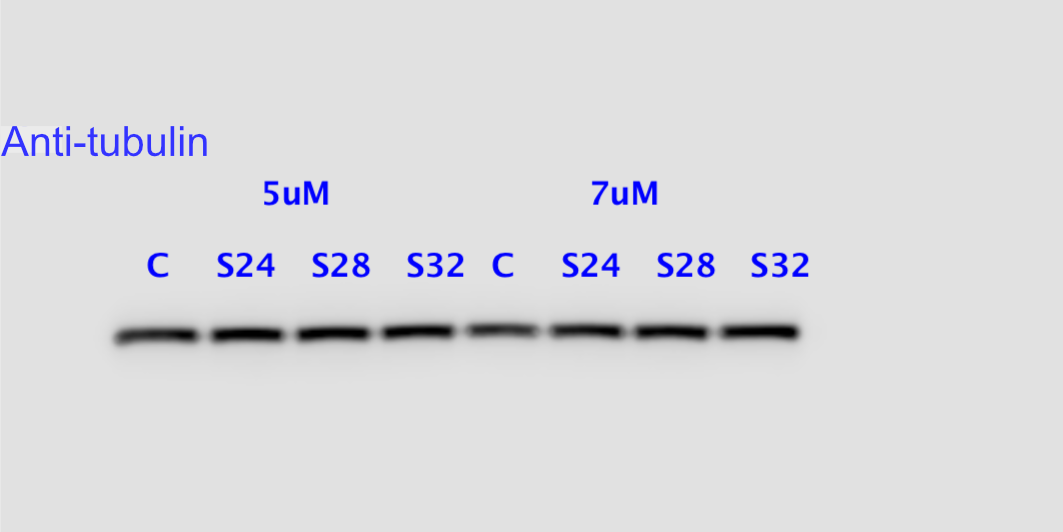


Fig 3 b.


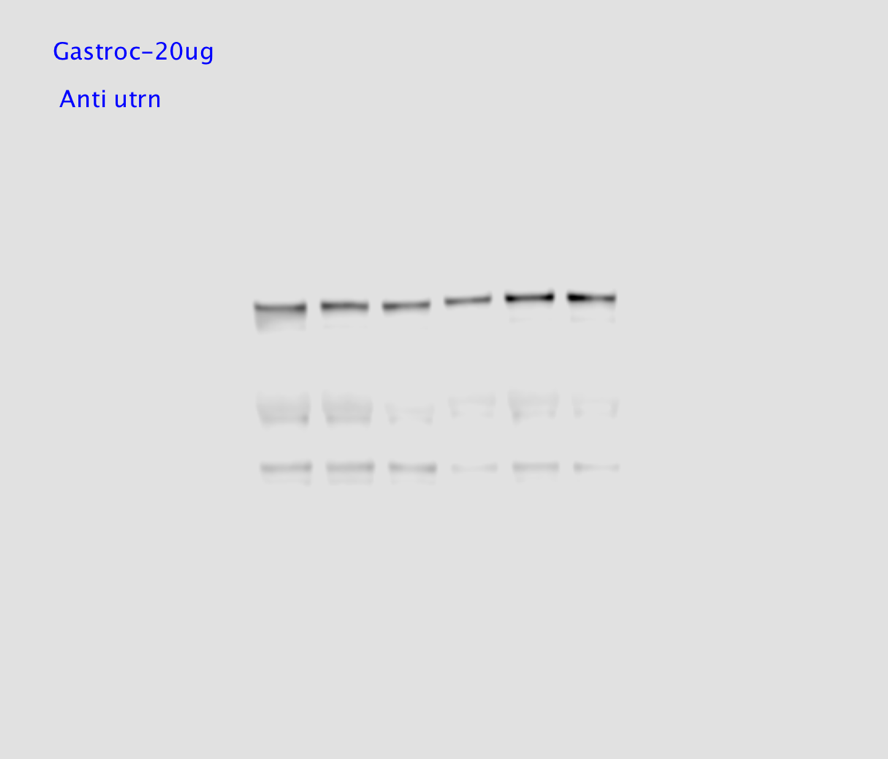


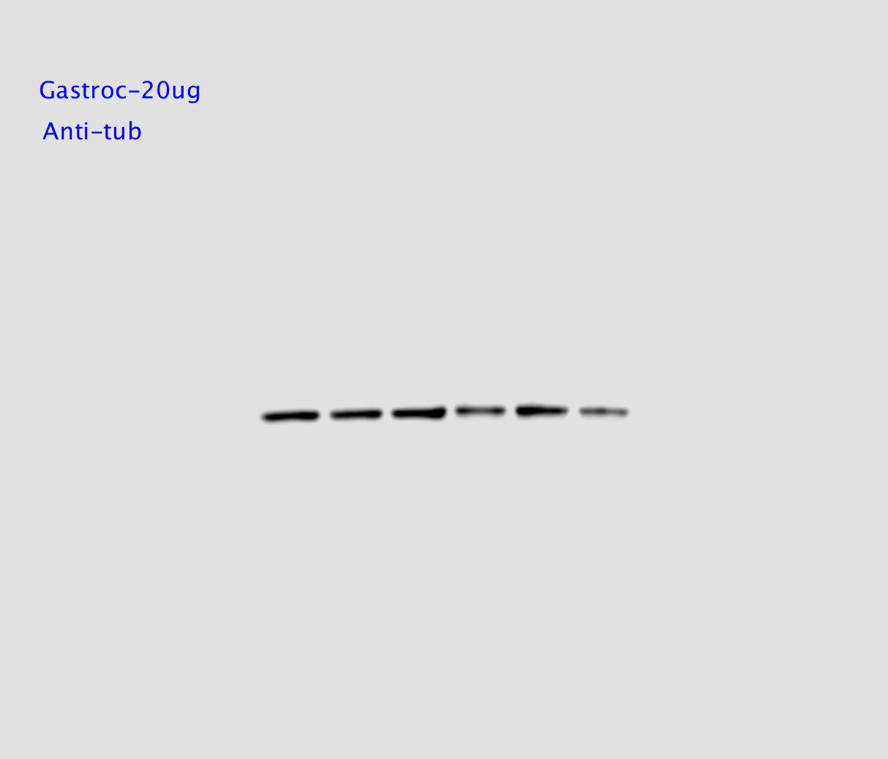


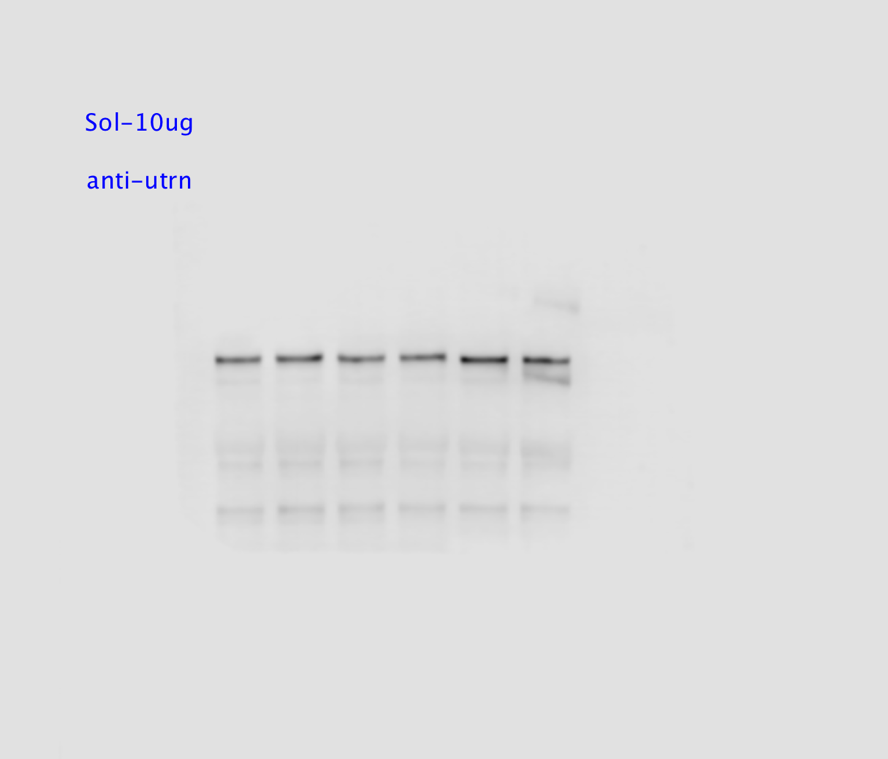


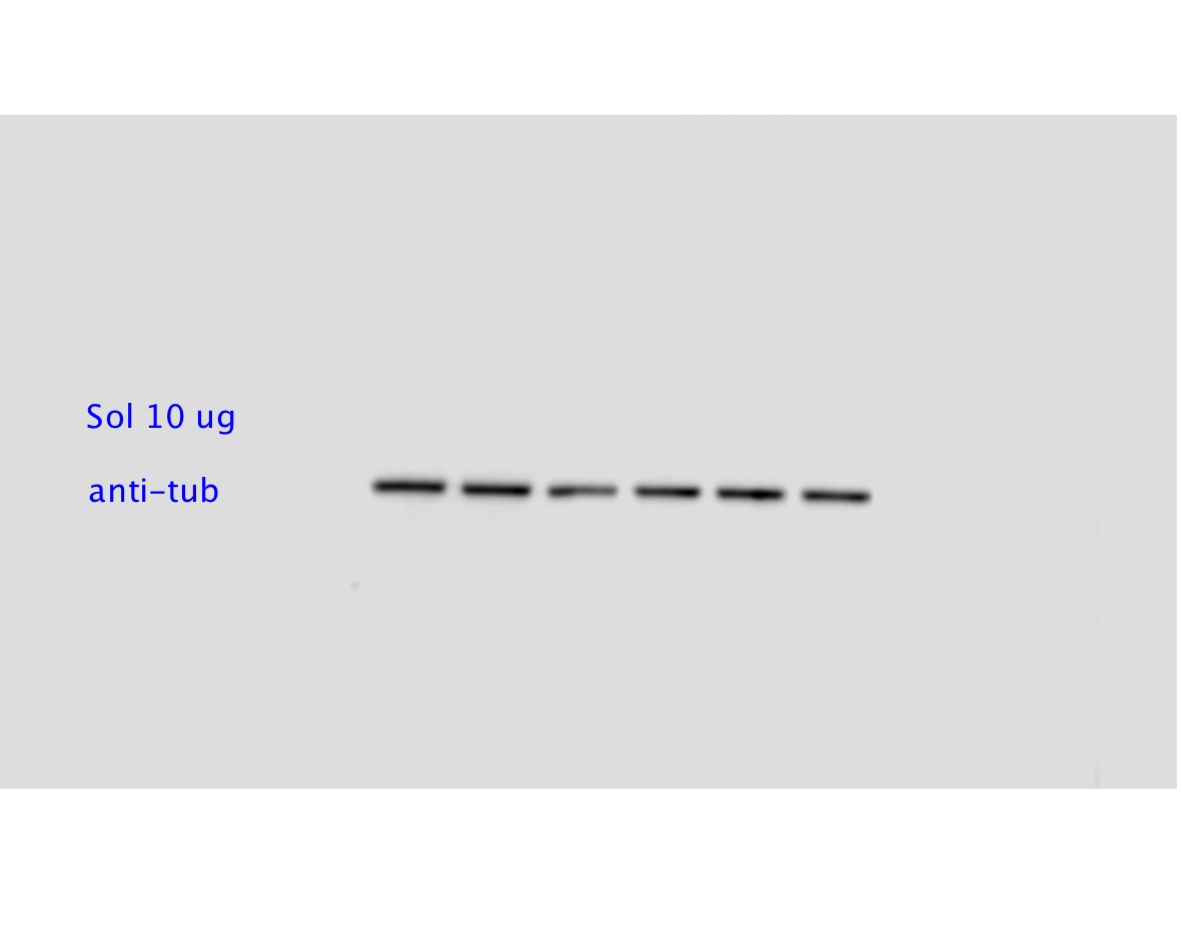


Supplementary figure 1


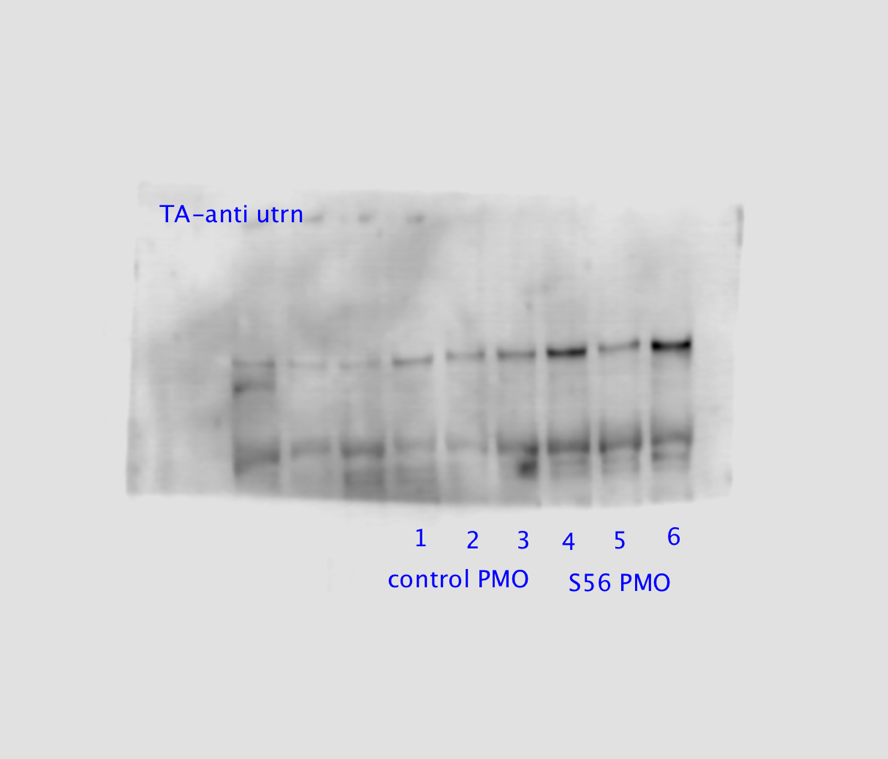


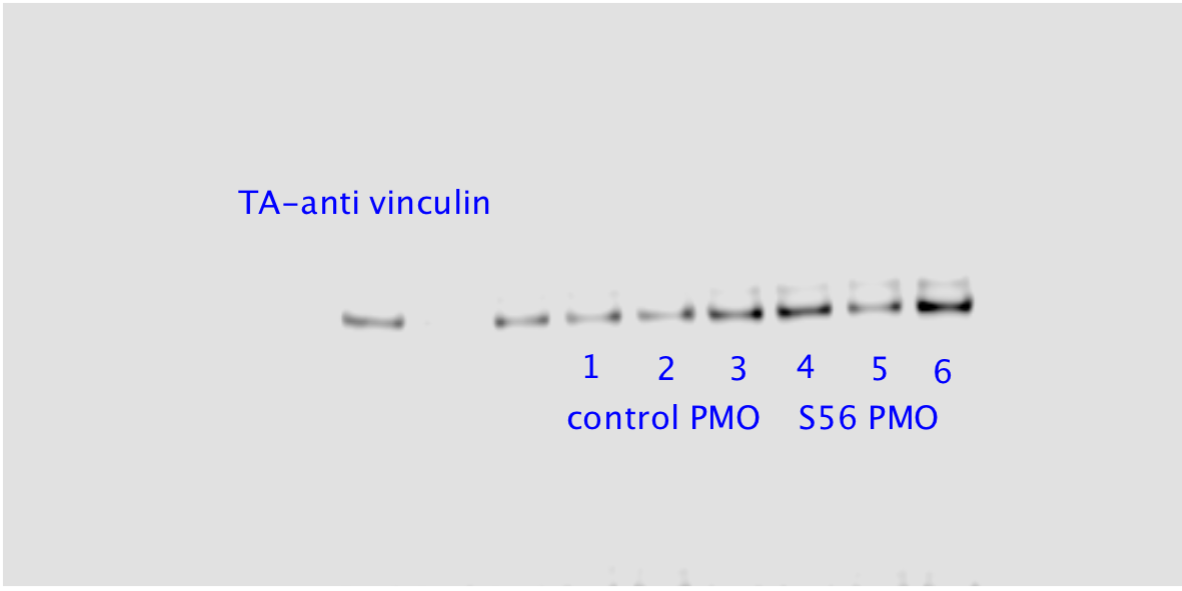


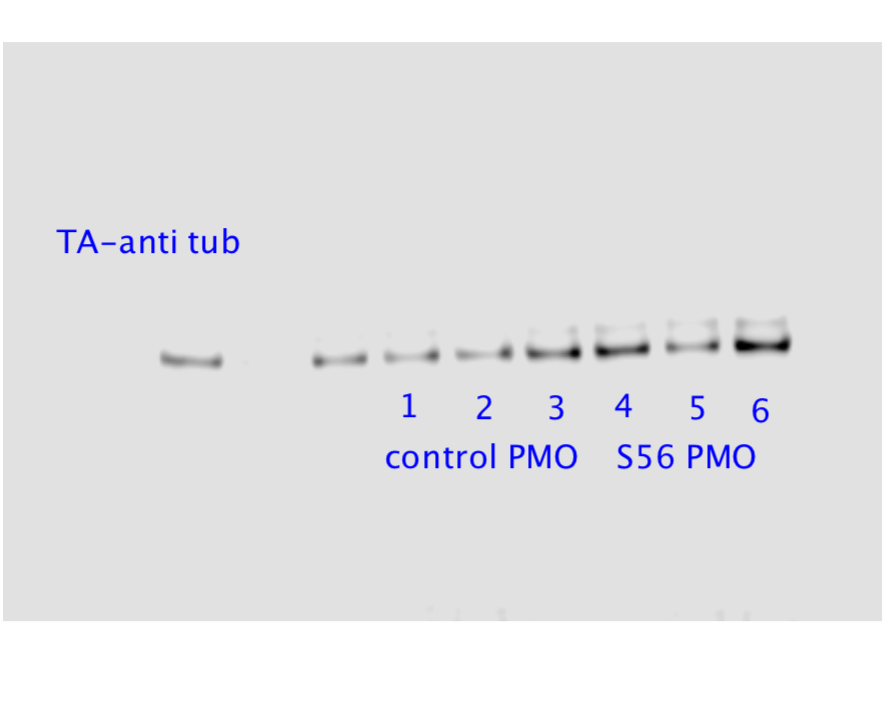


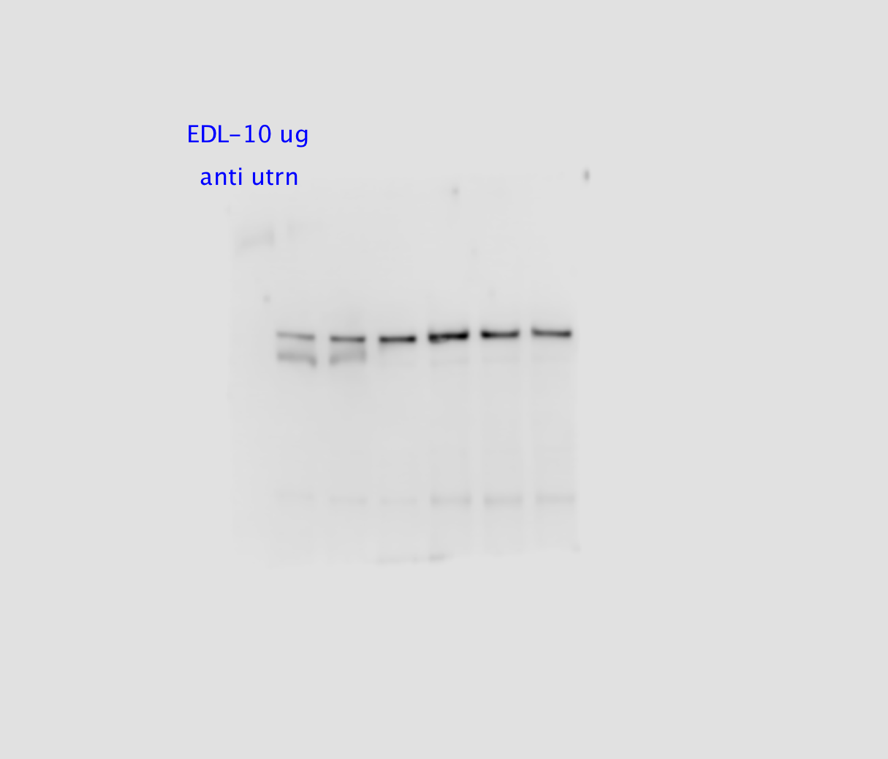


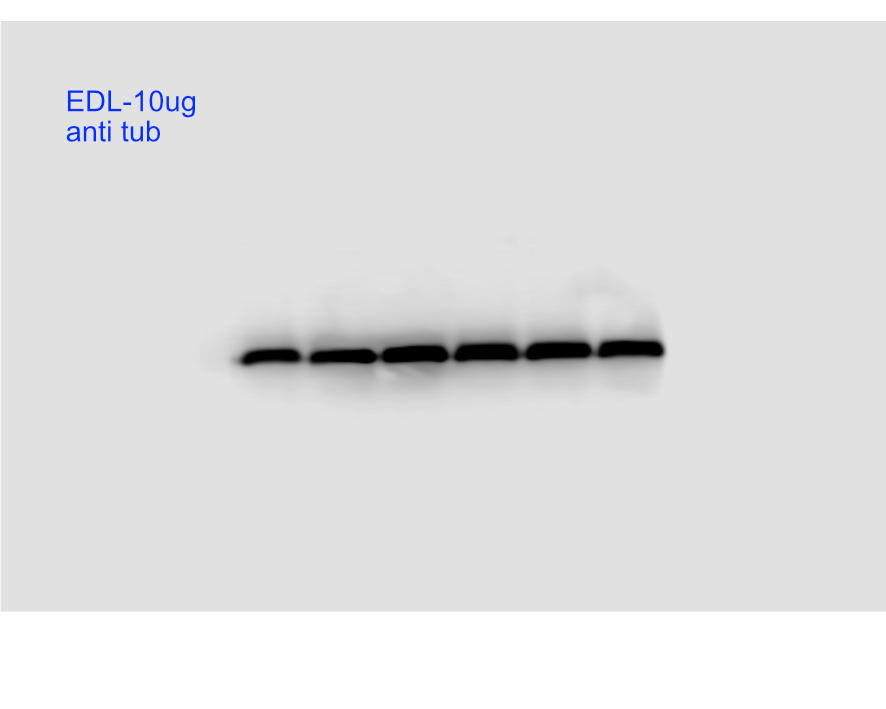


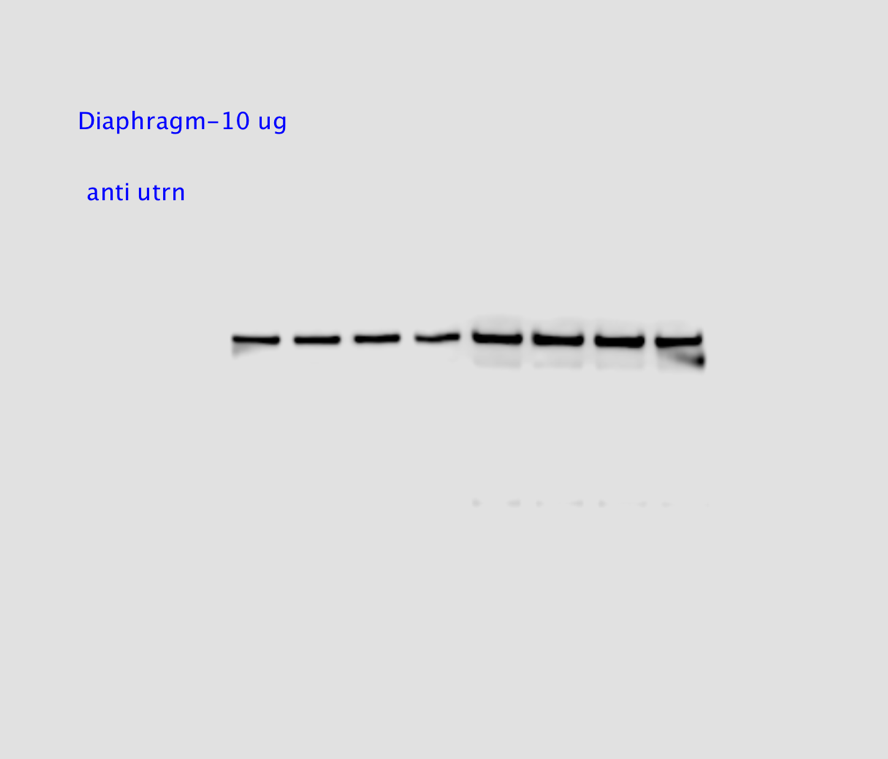


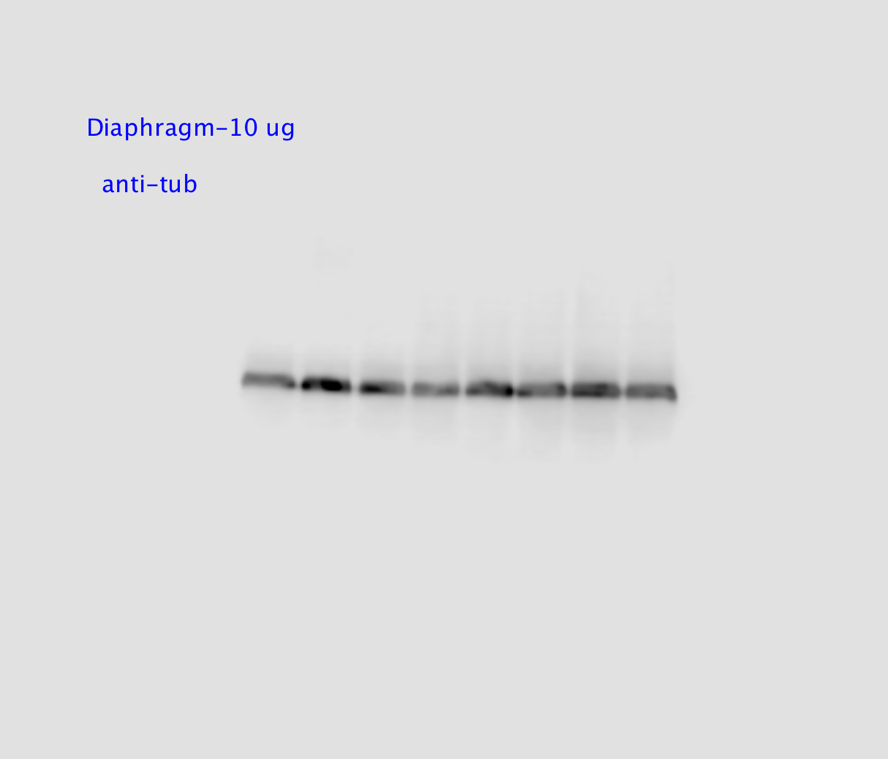


Supplementary Fig 3


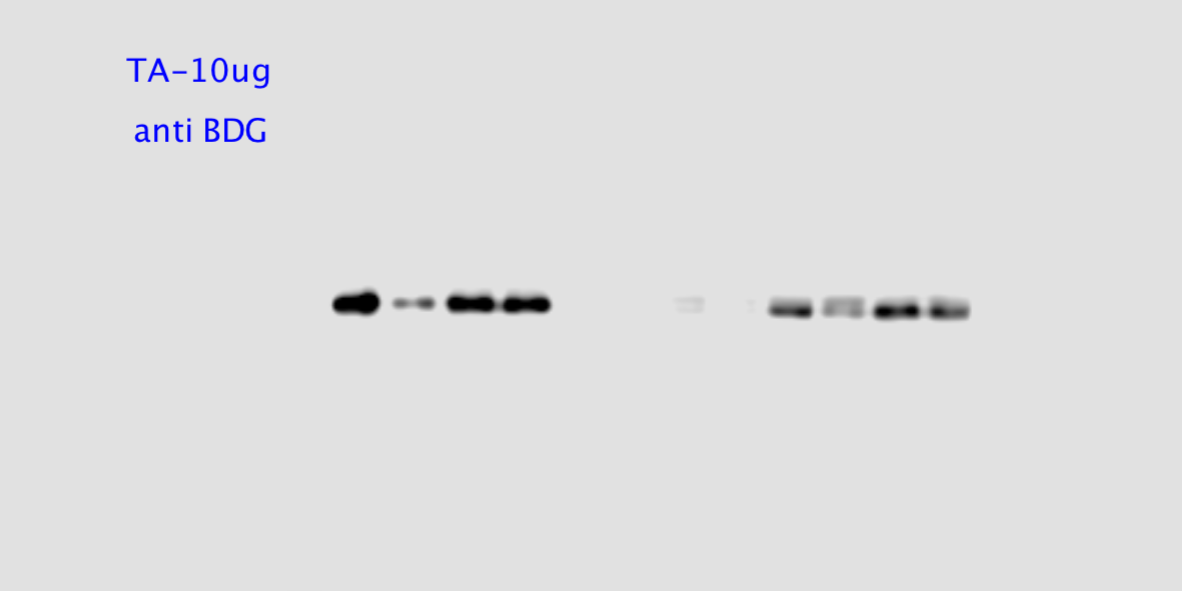


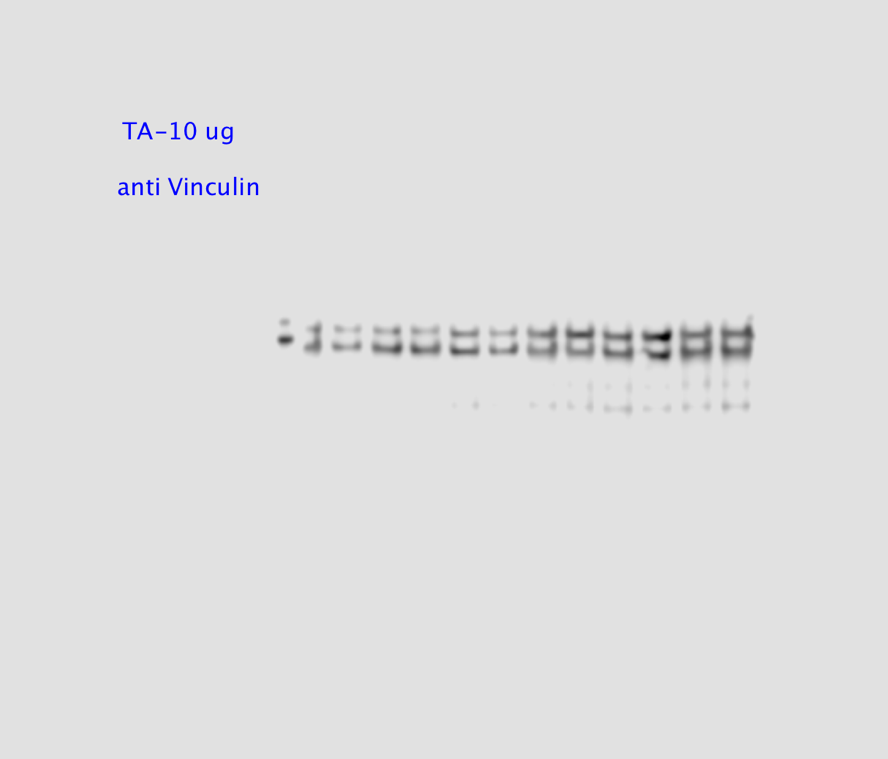


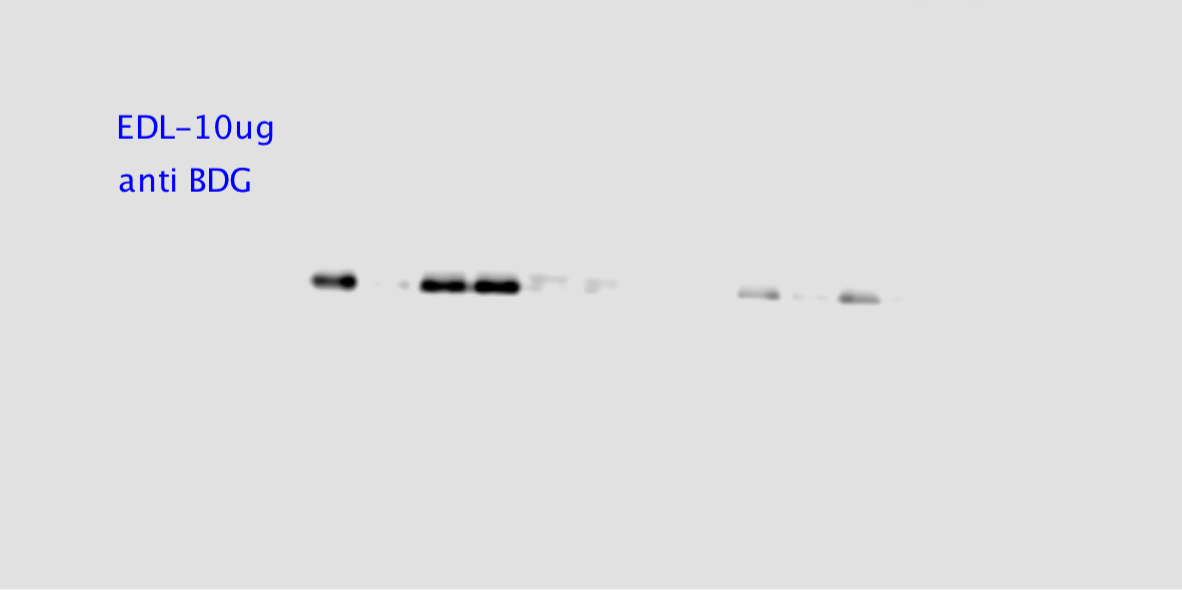


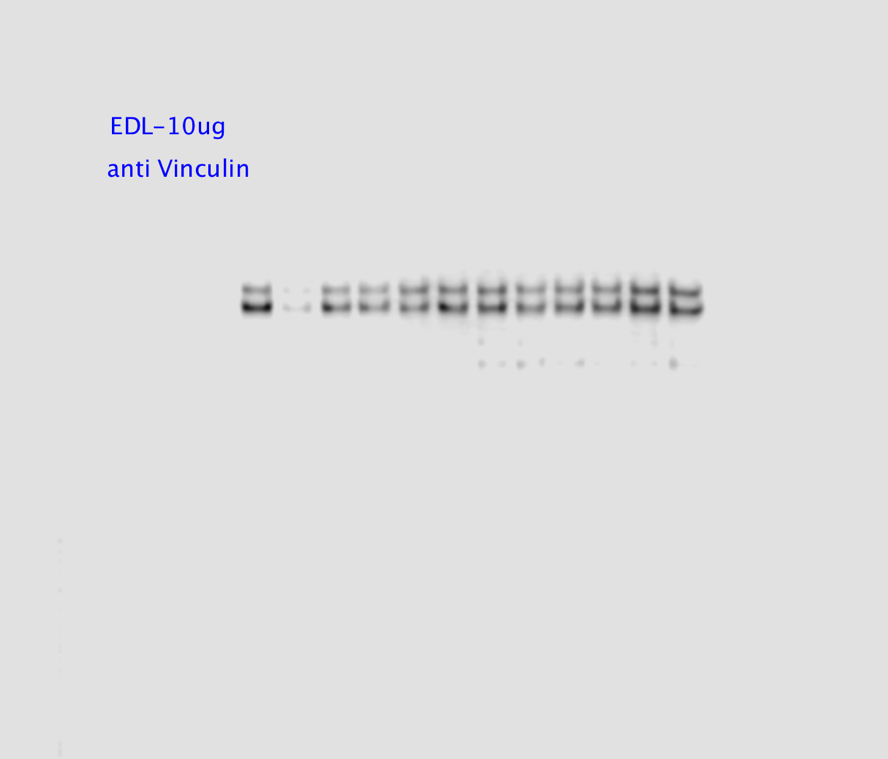

Supplement: Supplementary file 1 — Supplementary Information. [file 41598_2020_76338_MOESM1_ESM.docx]
